# Trait-based prediction of disease-vector mosquito invasion potential

**DOI:** 10.1371/journal.pntd.0014538

**Published:** 2026-07-23

**Authors:** Rebecca Pabst, Carla A. Sousa, César Capinha

**Affiliations:** 1 Global Health and Tropical Medicine, GHTM, LA-REAL, Institute of Hygiene and Tropical Medicine, IHMT, NOVA University Lisbon, Lisbon, Portugal; 2 Centre of Geographical Studies, Institute of Geography and Spatial Planning, University of Lisbon, Lisbon, Portugal; 3 Associate Laboratory TERRA, Lisbon, Portugal; Egerton University, KENYA

## Abstract

Mosquito-borne diseases pose a growing global health threat, largely driven by the human-mediated introduction of vector species beyond their native regions. Although only a few mosquito species have historically established populations outside their native ranges, the number of such invasions has increased rapidly in recent decades. Once established, these non-native invasive mosquitoes are difficult to control, emphasizing the need for proactive identification before introduction occurs. Here, we present a framework to predict invasion potential for 184 mosquito species of medical importance based on their ecological, life-history, and macroecological traits. We compiled a comprehensive dataset of 26 traits characterizing each species and used random forest models to relate these traits to the probabilities of introduction into non-native regions (before and after 1950, marking the onset of widespread trade globalization) and establishment following introduction. Models achieved moderate to good predictive performance (AUC = 0.78–0.85) and revealed that species native to Asia and Australia, adapted to human-made breeding sites, and associated with high-precipitation and thermally extreme environments were consistently more likely to be introduced or establish in non-native regions. Among species with no known invasion history, we identified 24 with high predicted introduction probabilities, of which 17 also showed high establishment probabilities. These results demonstrate that invasion potential can be inferred from species traits and provide a quantitative basis for proactive surveillance and prioritization of species most likely to be introduced in the future.

## Author summary

Mosquito-borne diseases threaten more than half of the world’s population and cause over 700,000 deaths each year. Although only a small proportion of mosquito species can transmit these diseases, human activities such as trade and travel are increasingly introducing vectors into new regions. The establishment of these non-native invasive species has led to growing numbers of locally transmitted outbreaks in areas that previously, or in recent history, experienced little or no mosquito-borne disease transmission. Therefore, it is crucial to understand which species are most likely to be introduced into new regions and to identify those that should be prioritized for surveillance and prevention efforts. In this work, we linked species characteristics with invasion history to identify the factors associated with mosquito introduction and establishment in non-native regions. We found that species native to Asia and Australia, capable of exploiting human-made breeding sites, and associated with high-precipitation and thermally extreme environments showed the highest predicted probabilities of introduction and establishment. Using these relationships, we identified 24 species with high predicted invasion potential, including 17 that also showed high probabilities of establishment following introduction. These results demonstrate that invasion potential can be partially inferred from measurable species traits, providing a quantitative framework to support early-warning surveillance and prioritize species for monitoring before widespread establishment occurs.

## 1. Introduction

Mosquito-borne diseases are a major threat to global public health, causing more than 700,000 deaths annually and placing over half of the world’s population at risk of infection [1]. This risk is largely attributed to a small group of competent vector species capable of transmitting pathogens such as dengue, Zika, chikungunya, malaria, and West Nile virus [2,3]. Historically, many of these species maintained relatively restricted distributions. However, in recent decades, the range of several mosquito vectors has expanded at an unprecedented pace, due to human-mediated introduction to new regions. Of the approximately 184 mosquito species, from which human pathogens have been isolated from wild-caught females [4], by now 46 have already been introduced into regions outside their native ranges, with 29 confirmed as having established populations [5,6]. These introductions have expanded the species’ ranges, sometimes into new continents and other distant regions. In several instances, they have also enabled local transmission of mosquito-borne pathogens in areas previously considered free of these diseases or unsuitable for sustained transmission [7,8].

Despite receiving increasing attention, the drivers of mosquito introductions and establishment in non-native regions remain poorly understood. Some species, such as *Aedes aegypti*, have expanded globally since the 15^th^ century [9], while others, like *Aedes albopictus*, *Aedes japonicus* and *Anopheles stephensi*, emerged as invasive non-native species (*i.e.,* introduced and established populations outside their native range; cf., [10]) only in recent decades [11–13]. For many other mosquito species, however, there is no evidence that they were transported by humans or successfully established themselves following introductions. This is intriguing because, in several cases, such species have ecological traits, habitat use, or associations with humans that are broadly similar to those of invasive species [14]. Hence, a key question in vector ecology and prevention remains: why are some mosquito species being introduced and becoming established in non-native regions while others remain restricted to their native ranges? Shedding light on drivers of these differences would be of key relevance for supporting surveillance and introduction-prevention efforts, allowing to help identify species more likely to become introduced in the future. Similarly, in a global context where resources for surveillance are limited and the pool of known invasive species is likely incomplete [15], understanding these factors may also help identify introduced or invasive species that remain undetected.

In order to provide warning lists to guide preventive biosecurity policies, trait-based approaches have recently emerged as valuable tools to identify species more likely to become introduced and invasive (e.g., [16–18]). By integrating variables representing ecological and life-history traits and macroecological patterns into predictive frameworks, these approaches assess which species are more or less likely to enter the global invasion pathway, while also highlighting the traits mediating interspecific variation in invasion potential. For example, Pili et al. [17] developed a global trait-based model and showed that to predict species establishment, both life-history and macroecological traits were important predictors and show that invasion success concentrates in species combining ecological flexibility with high probability of human mediated transport. Similar trait-based assessments for disease vector mosquitoes would provide critical advances in anticipating future biological invasions and the emergence of vector-borne diseases to protect naïve human populations. Revealing which mosquito species are predisposed to introduction and establishment, would support surveillance planning, refine monitoring priorities and advance our understanding of how ecological specialization, climate tolerance, and human-mediated transport interact to shape global mosquito invasion risk. However, to date, such an assessment has been constrained by the lack of comprehensive trait data, with most compilations restricted to a subset of species of known medical importance [19,20].

Here, we take advantage of a newly compiled, extensive database covering 184 mosquito species capable of natural infection with human pathogens to perform a trait-based assessment of species introduction and invasion potential. The trait dataset integrates species ecological, life-history, and macroecological data and to our knowledge is by far, the most taxonomically comprehensive available. Thus, this dataset provides a unique opportunity to test which characteristics of species are associated with a higher or lower invasion potential. Specifically, we combined these data with data from a recent global assessment of mosquitos introduction records and non-native ranges [5] within a machine learning framework to: (i) identify species traits that predict their propensity for introduction and establishment in non-native regions and, (ii) assess which currently unintroduced or non-established species may pose future invasion risks.

## 2. Methods

### 2.1. Trait compilation

**Fig 1:**
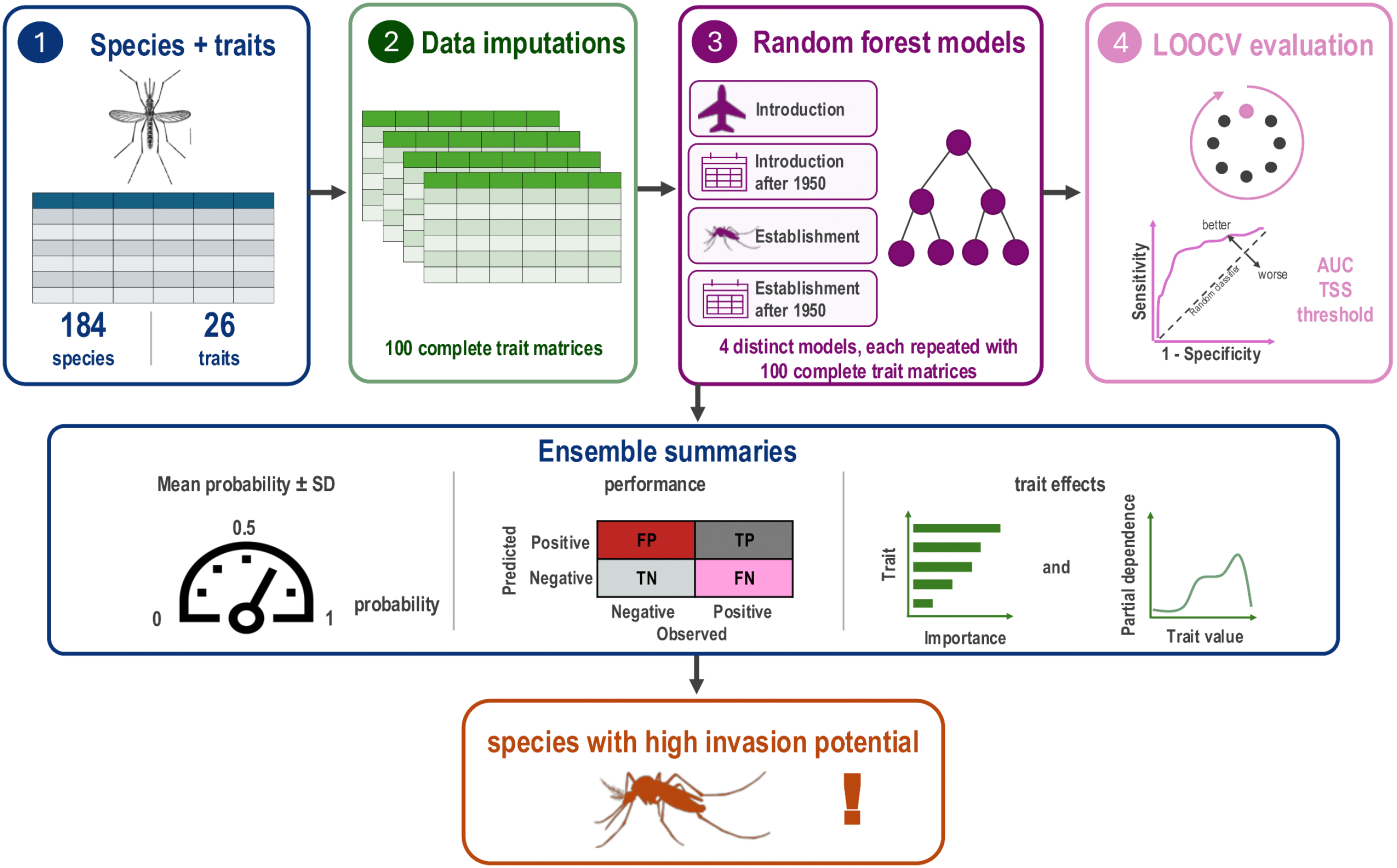
Overview of the analytical workflow used to identify mosquito species with high invasion potential. Species-level trait data were compiled for 184 species and 26 traits, and missing values were addressed using 100 imputed complete trait matrices. Random forest models were then fitted to predict introduction, introduction after 1950, establishment, and establishment after 1950. Model performance was evaluated using leave-one-out cross-validation, including AUC, TSS, and threshold-based metrics. Ensemble summaries were used to estimate mean invasion probabilities, classification performance, and trait effects, including variable importance and partial dependence. The workflow outputs a ranked set of species with high predicted invasion potential and is implemented in reproducible R code.

Our aim was to estimate the probability that mosquito vector species are introduced outside their native range and once introduced, establish self-sustaining non-native populations (Fig 1).

Species included in the analysis followed Pabst et al. [5] and were limited to 184 vector mosquitoes from which human pathogens were isolated from field-caught females, verified by at least two literature sources [4] or by one source plus a disease association recorded in Wilkerson et al. [21]. The list of assessed species is provided in S1 Appendix, the introduction and establishment status of species included in the modelling is provided Table A in S2 Appendix.

The propensity of mosquito species to become introduced and established in non-native regions is expected to depend on multiple ecological and macroecological factors [22]. Introduction likelihood should be influenced by intrinsic traits mediating associations with human-traded commodities and transport pathways, such as the use of human-made breeding sites including used tires and ornamental plants, which are recognized pathways for globally invasive species such as *Aedes aegypti* and *Ae. albopictus* [23]. Establishment success is also expected to depend on traits influencing survival during transport and persistence after arrival, including desiccation resistance, environmental tolerances, climatic adaptability, and the prevalence of viable propagules (*i.e.,* mosquito life stages capable of founding new populations, such as eggs, larvae, or gravid females) upon arrival [24].

To capture these different dimensions of invasion potential, we assembled 26 ecological, life-history, and macroecological traits for these species (Table B in S2 Appendix, S1 Appendix). Ecological and life-history traits describe species-specific intrinsic attributes expected to influence transport, survival during transit, and establishment, including breeding habitat, use of human-made breeding sites, egg desiccation resistance, oviposition strategy and host-use patterns.

Macroecological traits represent the native-range context of each species, including native-range size, climatic limits, and broad biogeographic origin. These predictors were included because they may capture both environmental tolerances and historical exposure to external factors like human-mediated transport pathways and opportunities for introduction and thus serve as proxies of invasion history as well as current and past propagule pressure.

We focused on variables that are relatively stable across environmental gradients and for which information was available for most species, thereby minimizing data gaps and ensuring general applicability. Ecological and life-history traits were compiled through literature search, and each species-trait combination was referenced to a specific literature source (S1 Appendix). Macroecological variables were derived from native-range distribution data and spatial environmental layers (CHELSA; [25,26]) or obtained from specialized sources like species distribution from Wilkerson et al. [21] and blood meal hosts from Soghigian et al. [27]. The rationale for including these derived traits and details on the procedures for their collection are described below.

#### 2.1.1. Country wide distribution data and native range delineation.

We delineated native ranges from country-level distribution records to characterize each species’ natural ecological context and assess the climatic conditions occupied (see below). Native geographic distribution may also influence invasion likelihood, as species occupying broad native ranges, particularly those overlapping regions of intense trade and connectivity, are expected to experience greater exposure to human-mediated transport opportunities. Country-level distributions were obtained from Wilkerson et al. [21] and the Walter Reed Biosystematics Unit [28]. For each species, all countries identified as non-native in the comprehensive compilation of Pabst et al. [5] were removed from these distributions. The remaining countries were treated as the species’ native range and were checked for spatial consistency with additional published distribution information (*e.g.,* [29,30]). From these native ranges we calculated native-range area (sum of constituent country areas in km^2^) and coded native continent(s) as binary variables. These variables were used to describe broad biogeographic origin and native-range extent.

#### 2.1.2. Climate data.

Our trait-based predictive framework requires understanding the climatic tolerances that determine a species’ survival, development, and reproductive cycles. Temperature constrains processes, such as gonotrophic cycle duration, body size, and fecundity [31,32], while precipitation shapes larval habitat availability by influencing standing water body formation and habitat persistence [33]. To characterize each species’ climatic niche, we extracted native-range climate values from CHELSA v2.1 layers [25,26] at 30 arc-second resolution (~1 km at the equator). We assembled occurrence records (1980–2024) from GBIF [34] (via the ‘rgbif’ package; Chamberlain et al. 2012) and from VectorMap [28], two global sources of mosquito data. We then cleaned the records for coordinate accuracy, duplicates, and outliers using the ‘CoordinateCleaner’ package [35]. Points were restricted to one per grid cell within the species’ native range and overlaid with monthly maximum and minimum temperature, and precipitation layers, to calculate annual averages (temperature) and totals (precipitation). Climatic tolerance thresholds were defined by the 2.5^th^ and 97.5^th^ percentiles to minimize errors from anomalous records caused by errors in georeferencing or species identification.

#### 2.1.3. Host preference.

Mosquitoes’ dispersal potential and habitat use are shaped by the availability of their preferred blood meal hosts. Generalist, anthropophilic and mammal-feeding species often occupy urban or peri-urban environments [36], facilitating unintentional human transport, whereas specialised ornithophilic species are typically associated with forested or wetland settings [37]. To include this in our model we obtained blood meal host data from Soghigian et al. [27], who compiled molecular blood meal analyses quantifying feeding proportions on mammals, birds, reptiles, and amphibians, providing a standardized host use metric across taxa.

### 2.2. Imputation of missing data

Despite our efforts of data compilation, several species still had incomplete trait information. To ensure the reliability of model estimates, we retained only species with at least 75% trait data completeness and at least three unique occurrence points, resulting in 169 modelled species from the initial pool of 184. For kept species, the data gaps were imputed using the ‘missForest’ algorithm [38], which iteratively predicts missing values via random forest. To account for imputation uncertainty, we repeated the imputation 100 times, producing 100 complete datasets. Imputation accuracy was evaluated using out-of-bag (OOB) error estimates (normalized root mean square error (NRMSE) for continuous variables; proportion falsely classified (PFC) for categorical variables).

### 2.3. Modeling framework and response variables

We used a random forest (RF) modeling framework to estimate the probability of introduction and establishment of mosquitoes that transmit diseases to humans, based on the fully imputed trait datasets. RF is a nonparametric ensemble learning method that handles continuous, categorical, and partially redundant predictors with high robustness to overfitting [39]. The algorithm builds multiple decision trees from bootstrap samples and aggregates their predictions, resulting in stable, nonlinear models with strong predictive performance. RF models perform well with sparse or noisy ecological data and offer interpretable measures such as variable importance and partial dependence plots [39,40]. These strengths have led to their widespread application in ecological and epidemiological studies [41–44].

Our models require two sets of input components: A binary response variable representing a species’ introduction or establishment status, and a set of predictor variables describing mosquito ecological, life-history, and macroecological traits. Four binary response variables were assembled: introduction outside the native range, introduction after 1950, establishment outside the native range, and establishment after 1950. In each model the binary response variable was coded as “1” when species met the criterion and “0” otherwise. Introduction and establishment dates were derived from Pabst et al. [5,6] and are listed in Table A in S2 Appendix. All 169 retained species were included in each model. The introduction model included 46 species coded as introduced, and the establishment model included 29 species coded as established. Restricting the analyses to post-1950 invasion events resulted in 41 and 26 species being classified as introduced and established, respectively.

The year 1950 was used as an additional temporal threshold because it broadly marks the onset of the post-World War II ‘Great Acceleration’ in global trade, transportation, and human connectivity, a period associated with major increases in the number and diversity of biological invasions [45–47]. Because invasion processes are strongly shaped by human-mediated transport dynamics, trait associations inferred from older introduction events may not fully represent the mechanisms driving contemporary invasions. The post-1950 models were therefore developed to better capture the transport and introduction dynamics operating under current globalization conditions and to provide invasion predictions more directly relevant to present-day and near-future introduction processes.

### 2.4. Variable selection

To minimize redundancy and collinearity among predictors, pairwise Pearson correlations were calculated for numerical variables, removing one of each pair with |r| > 0.7 [48]. Multicollinearity was further evaluated using the variance inflation factor (VIF) with the ‘vifstep’ function from the ‘*usdm’* R package [49], keeping only variables with a VIF < 5 [50]. As a result, we excluded the variables: Oviposition in human-made breeding sites and proportion of avian blood meal hosts. Following this process, in each model we used the same non-redundant combination of continuous, binary, and categorical predictors. The correlation structure of the final numeric variables is shown in Fig A in S2 Appendix.

In addition to models including the full set of ecological, life-history, and macroecological traits, we also fitted models excluding biogeographic predictors to assess the consistency of species-specific traits as drivers of introduction and establishment. In the main text, we present only the models including the full predictor set, as these achieved higher predictive performance (see Results) and are expected to better represent historical and contemporary propagule pressure conditions. The results of models using the reduced set of predictors are provided in the S2 Appendix.

### 2.5. Model fitting and cross-validation

For each response variable, the 100 imputed datasets produced in section 2.2 were used to train 100 separate RF models having the same parameters, implemented in the ‘*ranger’* package [51]. Following standard practice, each model was built with 1,000 trees to ensure prediction stability. Each tree used four randomly selected predictors at each split, following the common rule of using the rounded square root of the total number of predictor variables in classification models. A minimum node size of five was specified to reduce overfitting and to ensure that each terminal node contained a sufficient number of observations for stable mean estimates [52].

Model performance was evaluated through leave-one-out cross-validation (LOOCV) [53], in which each species was iteratively withheld from the training set, the model is fitted to the remaining species, and predictions are generated for the excluded species. This was repeated until every species had been left out once, resulting in a full set of out-of-sample predictions for each imputed data set and response variable. This approach is robust and mimics a real-world situation where the invasion potential of a species is assessed based on what has been observed for other species.

Model performance was evaluated by comparing predicted probabilities with observed outcomes across all species, using the area under the receiver operating characteristic curve (AUC; [54]) as a measure of discrimination ability. AUC ranges from 0 to 1, with values of 0.5 indicating discrimination no better than random expectation, whereas values closer to 1 indicate stronger discriminatory performance. While AUC is a robust, threshold-independent metric for model evaluation and comparison, it does not indicate the probability cutoff at which classification performance is maximized.

In our context, identifying such a threshold is important because it allows us to identify which species were correctly or incorrectly classified by the models. To address this, we used the True Skill Statistic (TSS; [55]), which identifies the probability threshold that maximizes classification accuracy by balancing sensitivity and specificity. The optimal threshold of each RF model was identified by maximizing TSS with the function ‘ecospat.max.tss()’ from the ‘ecospat’ package [56]. Species with predicted probabilities above this threshold were classified as predicted positives, allowing identification of true positives, true, negatives, false positives, and false negatives and associated metrics (sensitivity and specificity).

For each species and response variable, predicted probabilities were averaged across the 100 replicate RF models to obtain ensemble mean predictions. The standard deviation (SD) across replicates was used as a measure of uncertainty associated with imputation and model stochasticity. Performance metrics, optimal TSS thresholds, confusion-matrix counts, were summarized in the same way, using means and standard deviations across the 100 replicates.

### 2.6. Variable importance and trait effects

For each of the four response variables, we additionally ran 100 full RF models with all species to calculate contributions of the predictors using permutation-based importance scores, which represent the decrease in model accuracy after shuffling each variable [39]. Variable importance scores were averaged across replicates to obtain stable ensemble estimates. SD across repetitions was used to express uncertainty in variable ranking. Variables that showed consistent influence (importance ≥ 0.01 across models) were further explored [52] using partial dependency plots generated from the averaged ensemble predictions, using the FeatureEffect$new() function in the ‘iml’ package [57].

### 2.7. Identifying species with high predicted invasion potential

We used ensemble leave-one-out predictions of the 100 RF model repetitions to identify species whose predicted probabilities were high relative to their currently observed invasion status.

First, we identified species not currently recorded as introduced but whose mean predicted introduction probability exceeded the ensemble mean optimal TSS threshold for the introduction model. These species represent false positives in the statistical sense: they are currently observed as non-introduced but are predicted by the model to have trait profiles similar to introduced species. We interpret them as species with high predicted introduction potential.

(We then cross-checked this list of species with predictions from the establishment model. Species whose mean predicted establishment probability also exceeded the ensemble mean optimal TSS threshold were classified as species with high predicted invasion potential because they exhibited simultaneously high predicted introduction and establishment probabilities. For readability, we refer to these species as “species with high predicted invasion potential”.

We also identified false negatives, defined as species with known introduction or establishment records but predicted below the corresponding TSS threshold, to clarify where the models failed to identify known invaders.

All analyses were performed in R [58,59], with scripts and reproducible code provided in the S3 Appendix.

## 3. Results

### 3.1. Model performance

A comprehensive dataset was compiled with sufficient data for 169 mosquito species characterized by 24 ecological, life-history and macroecological variables, including eight continuous, three categorical and thirteen one-hot encoded traits. Missing values were imputed with low error, averaging <0.01% for continuous traits and 3.3% for categorical traits.

**Fig 2:**
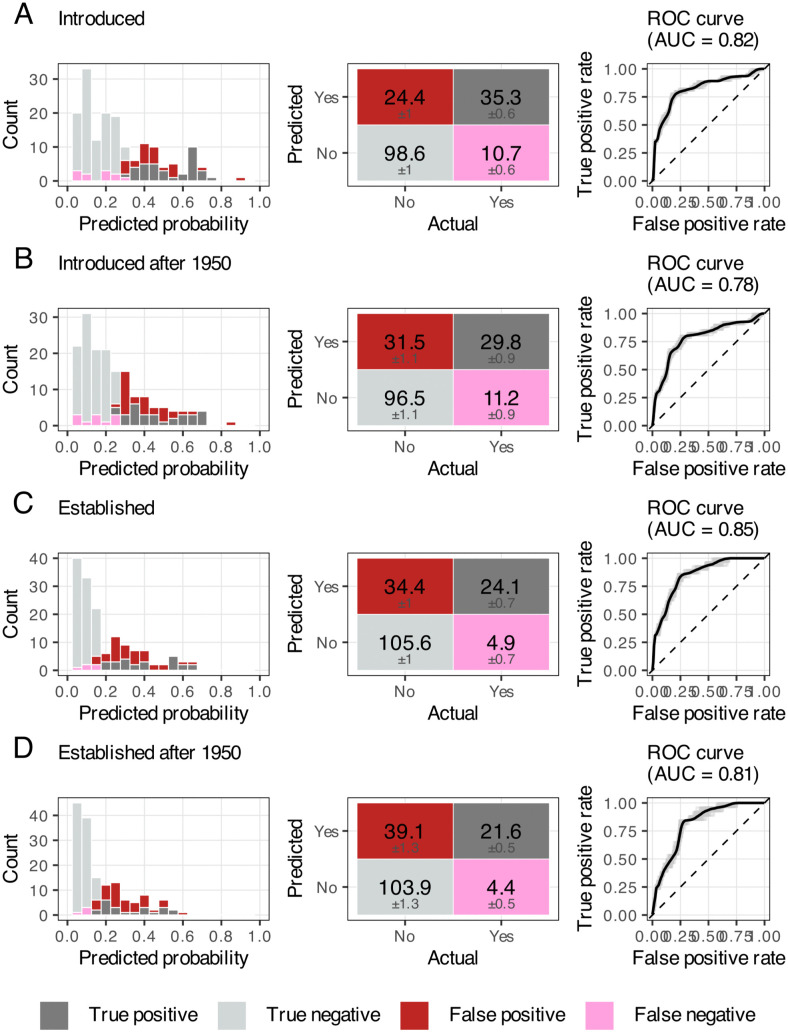
Leave-one-out cross-validation performance of random forest models predicting the introduction and establishment probabilities of mosquito species using the full set of trait variables. Panels show results for models predicting probabilities of (a) introduction (AUC = 0.82), (b) introduction after 1950 (AUC = 0.78), (c) establishment (AUC = 0.85), and (d) establishment after 1950 (AUC = 0.81). For each model, panels show from left to right, predicted probability distributions, mean ± SD confusion matrices, and ROC curves across 100 repetitions, with the average ROC curve shown in black. Red indicates false positives and pink indicates false negatives.

The random forest models showed moderate to good ability to distinguish species introduced or established in non-native regions and those without known invasion history. Across the 100 imputed datasets, mean AUC values ranged from 0.78 for introductions after 1950 to 0.85 for establishment, with little variation among replicates (SD < 0.004; Table 1, Fig 2). The introduction models exhibited higher specificity than sensitivity, suggesting stronger performance in identifying non‐introduced species, while the establishment models displayed the opposite pattern, reflecting improved recognition of species capable of successful establishment.

**Table 1:** Leave-one-out cross-validation random forest model performance metrics for the models including the full set of trait variables. Values show mean ± SD across 100 model repetitions for models predicting introduction, introduction after 1950, establishment and establishment after 1950.

Response	AUC	OOB error	Optimal TSS threshold	Sensitivity	Specificity
Introduced	0.82 ± 0	0.14 ± 0	0.30 ± 0.01	0.78 ± 0.02	0.80 ± 0.02
Introduced after 1950	0.78 ± 0	0.15 ± 0	0.26 ± 0.03	0.76 ± 0.06	0.76 ± 0.05
Established	0.85 ± 0	0.11 ± 0	0.17 ± 0.01	0.85 ± 0.02	0.75 ± 0.02
Established after 1950	0.81 ± 0	0.11 ± 0	0.16 ± 0.01	0.84 ± 0.01	0.73 ± 0.02

Several species without current introduction or establishment records were predicted above the optimal TSS thresholds (Fig 2, red), whereas some known introduced or established species fell below these thresholds (Fig 2, pink). Following the classification approach described in section 2.7, we use these mismatches to identify species with high predicted invasion potential and known invaders that were not recovered by the models; these species are reported in section 3.7.

### 3.2. Variable importance and partial dependency plots

**Fig 3:**
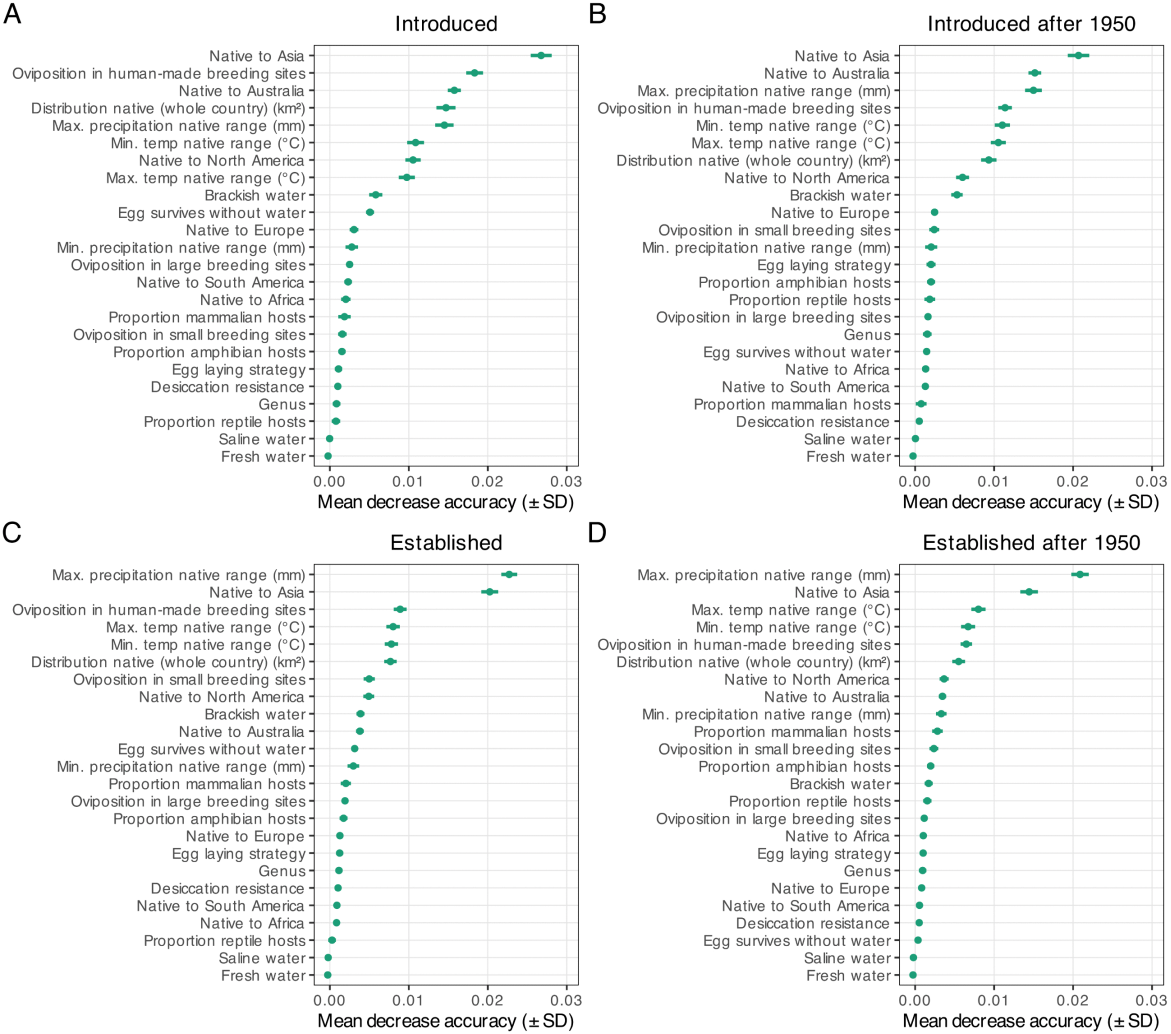
Relative importance of predictors in random forest models including the full set of trait variables. Points show mean decrease in accuracy ± SD across 100 model repetitions. Higher values indicate greater predictor importance for (a) introduction, (b) introduction after 1950, (c) establishment, and (d) establishment after 1950.

**Fig 4:**
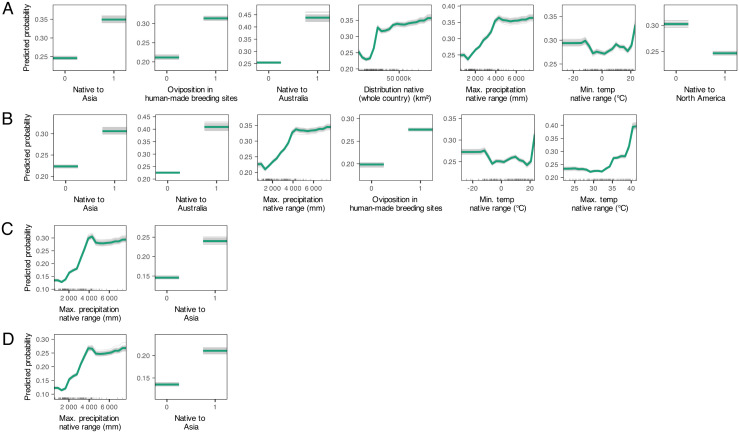
Partial dependence plots from the full random forest models including the full set of trait variables. Plots show the marginal effects of predictors with importance ≥ 0.01 on predicted probabilities. Grey lines show individual model repetitions and green lines show the average across 100 repetitions for (a) introduction, (b) introduction after 1950, (c) establishment, and (d) establishment after 1950.

Permutation importance indicated that native origin in Asia had the highest relative importance for predicting introduction probability, followed by oviposition in human-made breeding sites, native origin in Australia, native-range area, maximum precipitation, minimum temperature, and native origin in North America (Fig 3A). Partial dependence plots showed higher introduction probabilities for species native to Asia or Australia, species using human-made breeding sites, species with broader native distributions, and species associated with regions of high maximum precipitation (Fig 4A). In contrast, species native to North America showed lower introduction probabilities. The relationship with minimum temperature was non-linear, with higher introduction probabilities associated with species occurring in both cold and warm minimum-temperature regimes, whereas species from intermediate temperature ranges were less likely to be introduced. For introductions after 1950, the overall pattern was similar, but the relative importance of climatic predictors was more prominent (Fig 3B). Species native from Asia, using human-made breeding sites and native in regions with higher maximum precipitation and maximum temperature had higher predicted probabilities of post-1950 introduction (Fig 4B).

For establishment, maximum precipitation within the native range showed the highest relative importance in both the all-time and post-1950 models, followed by native origin in Asia (Fig 3C-3D). Partial dependence plots indicated that establishment probability increased with higher native-range maximum precipitation, with species from regions experiencing high rainfall maxima more likely to resemble known established invaders (Fig 4C-4D). Establishment probabilities were also higher for species native to Asia.

Models excluding biogeographic predictors showed similar predicted probabilities and rankings of important variables. However, their performance was generally lower than that of the models with the full set of traits (AUC = 0.76-0.82; S2 Appendix).

### 3.3. Predicted invaders and discrepancies

The introduction model identified 24 mosquito species without known invasion history, but with ensemble probabilities above the optimal TSS threshold, and whose traits therefore are consistent with unintentionally transported and introduced species (Table 2). These false-positive species share ecological profiles of known invaders, including human altered environments, geographic origin, and climatic characterization of their native range, underscoring their potential as emerging invaders warranting targeted surveillance. Seventeen of these species also exceeded the establishment threshold and are therefore interpreted as species with high predicted invasion potential. Their attributes are consistent with species that also established non-native populations. On the other hand, species exceeding the introduction threshold but not the establishment threshold may possess traits favoring transport and introduction, but not long-term establishment under non-native ecological or climatic conditions.

**Table 2:** Ensemble-predicted probabilities for false-positive species from the models including the full set of trait variables. False positives are species with no recorded positive invasion status for a given response variable but with ensemble mean predicted probabilities above the corresponding optimal TSS threshold. Values show ensemble mean predicted probabilities ± SD across 100 model repetitions for introduction, introduction after 1950, establishment and establishment after 1950. NA indicates that the species was not classified as a false positive for that response variable. Species in bold had above threshold predictions across all four models and are interpreted as having high predicted invasion potential. * indicate predictions exceeding the highest predicted probability among true positives for that response variable.

Species	Introduced	Introduced post-1950	Established	Established post-1950
***Culex vishnui***	0.89 ± 0.01*	0.85 ± 0.01*	0.58 ± 0.01	0.51 ± 0.01
***Anopheles culicifacies***	0.70 ± 0.01	0.56 ± 0.01	0.41 ± 0.01	0.42 ± 0.01
***Aedes lineatopennis***	0.57 ± 0.01	0.52 ± 0.01	0.39 ± 0.01	0.36 ± 0.01
***Culex univittatus***	0.57 ± 0.02	0.54 ± 0.01	0.44 ± 0.01	0.38 ± 0.01
***Anopheles amictus***	0.56 ± 0.01	0.52 ± 0.01	0.26 ± 0.01	0.26 ± 0.01
***Culex theileri***	0.55 ± 0.01	0.50 ± 0.01	0.41 ± 0.02	0.37 ± 0.01
***Mansonia septempunctata***	0.50 ± 0.01	0.51 ± 0.01	0.32 ± 0.01	0.31 ± 0.01
***Culex torrentium***	0.46 ± 0.01	0.44 ± 0.02	0.37 ± 0.02	0.36 ± 0.01
***Anopheles hyrcanus***	0.45 ± 0.01	0.37 ± 0.02	0.34 ± 0.01	0.28 ± 0.01
***Culex perexiguus***	0.45 ± 0.02	0.46 ± 0.02	0.27 ± 0.01	0.25 ± 0.01
***Culex poicilipes***	0.45 ± 0.01	0.38 ± 0.02	0.24 ± 0.01	0.22 ± 0.01
*Anopheles rufipes*	0.43 ± 0.02	0.31 ± 0.01	NA	NA
***Anopheles claviger***	0.42 ± 0.01	0.29 ± 0.01	0.25 ± 0.01	0.24 ± 0.01
***Culex nigripalpus***	0.42 ± 0.02	0.42 ± 0.01	0.33 ± 0.01	0.26 ± 0.01
***Anopheles plumbeus***	0.42 ± 0.01	0.32 ± 0.01	0.33 ± 0.01	0.29 ± 0.01
***Culiseta inornata***	0.40 ± 0.01	0.42 ± 0.01	0.34 ± 0.01	0.33 ± 0.01
*Anopheles fluviatilis*	0.40 ± 0.01	0.31 ± 0.01	NA	NA
***Aedes geniculatus***	0.40 ± 0.01	0.29 ± 0.01	0.31 ± 0.01	0.27 ± 0.01
***Anopheles pseudopunctipennis***	0.37 ± 0.01	0.31 ± 0.01	0.17 ± 0.01	0.20 ± 0.01
*Coquillettidia linealis*	0.36 ± 0.01	0.36 ± 0.01	NA	NA
*Coquillettidia richiardii*	0.32 ± 0.01	NA	NA	NA
*Anopheles sergentii*	0.32 ± 0.01	0.30 ± 0.01	NA	NA
*Anopheles pulcherrimus*	0.31 ± 0.01	0.30 ± 0.01	NA	NA
*Anopheles quadrimaculatus*	0.31 ± 0.01	NA	NA	NA
*Aedes africanus*	NA	0.28 ± 0.01	0.29 ± 0.01	0.28 ± 0.01
*Aedes scutellaris*	NA	0.66 ± 0.01	0.48 ± 0.02	0.43 ± 0.01
*Aedes togoi*	NA	0.61 ± 0.01	NA	0.51 ± 0.01
*Anopheles coustani*	NA	0.41 ± 0.01	0.17 ± 0.01	NA
*Anopheles funestus*	NA	0.39 ± 0.01	0.32 ± 0.01	0.23 ± 0.01
*Anopheles nuneztovari*	NA	0.28 ± 0.01	0.23 ± 0.01	0.24 ± 0.01
*Culex restuans*	NA	0.29 ± 0.01	NA	0.16 ± 0.01
*Culex salinarius*	NA	0.27 ± 0.02	NA	NA
*Psorophora confinnis*	NA	0.29 ± 0.01	0.29 ± 0.01	0.30 ± 0.01
*Aedes argenteopunctatus*	NA	NA	0.17 ± 0.01	0.16 ± 0.01
*Aedes camptorhynchus*	NA	NA	0.21 ± 0.01	0.20 ± 0.01
*Aedes polynesiensis*	NA	NA	0.25 ± 0.01	0.22 ± 0.01
*Aedes scapularis*	NA	NA	0.20 ± 0.01	0.19 ± 0.01
*Aedes taeniorhynchus*	NA	NA	0.21 ± 0.01	0.17 ± 0.01
*Aedes triseriatus*	NA	NA	0.25 ± 0.01	0.24 ± 0.01
*Anopheles barbirostris*	NA	NA	0.48 ± 0.01	0.42 ± 0.01
*Anopheles litoralis*	NA	NA	0.24 ± 0.01	0.18 ± 0.01
*Culex annulirostris*	NA	NA	0.43 ± 0.02	0.42 ± 0.01
*Culex antennatus*	NA	NA	0.41 ± 0.01	0.26 ± 0.01
*Culex bitaeniorhynchus*	NA	NA	0.67 ± 0.01*	0.59 ± 0.01*
*Culiseta morsitans*	NA	NA	0.24 ± 0.01	0.24 ± 0.01
*Anopheles arabiensis*	NA	NA	NA	0.41 ± 0.01
*Culex sitiens*	NA	NA	NA	0.51 ± 0.01
*Psorophora ciliata*	NA	NA	NA	0.16 ± 0.01

Some species with known invasion histories were not correctly classified by one or more models (Table 3). The false negatives had confirmed introduction or establishment records but predicted probabilities below the corresponding optimal TSS thresholds, indicating cases where invasion history was not fully explained by the available ecological, life-history, and macroecological traits. Most false-negative species showed limited invasion success: seven out of the 15 species had been introduced but had no confirmed establishment, and six had established in only one or two regions. These cases suggest that many false negatives may reflect transient introductions or failure to establish self-sustaining populations, suggesting stochastic introduction events or influences from drivers beyond trait-based predictors, such as chance transport or climatic anomalies, or traits not captured in the dataset.

**Table 3:** Known invaders not captured by the models including the full set of trait variables. False negatives are species with confirmed introduction or establishment records, but ensemble mean predicted probabilities below the corresponding optimal TSS threshold. Values show ensemble mean predictions ± SD across 100 model repetitions for each response variable. NA indicates that the species was not classified as a false negative for that response variable.

Species	Introduced	Introduced_post-1950	Established	Established_post-1950
*Aedes atropalpus*	NA	NA	0.11 ± 0.01	0.10 ± 0.01
*Aedes japonicus*	NA	0.24 ± 0.01	NA	NA
*Aedes mcintoshi*	0.05 ± 0.01	0.04 ± 0.01	NA	NA
*Aedes triseriatus*	0.25 ± 0.01	NA	NA	NA
*Aedes vigilax*	NA	NA	0.17 ± 0.01	NA
*Anopheles crucians*	0.06 ± 0.01	0.05 ± 0.01	NA	NA
*Anopheles darlingi*	0.11 ± 0.01	0.10 ± 0.01	0.08 ± 0.01	0.06 ± 0.01
*Anopheles maculipennis*	0.20 ± 0.01	NA	NA	NA
*Anopheles pharoensis*	0.27 ± 0.01	0.13 ± 0.01	NA	NA
*Anopheles punctipennis*	0.20 ± 0.01	0.20 ± 0.01	NA	NA
*Culex coronator*	0.11 ± 0.01	0.13 ± 0.01	0.07 ± 0.01	0.09 ± 0.01
*Culex modestus*	NA	0.26 ± 0.01	NA	NA
*Culex tarsalis*	0.30 ± 0.01	0.25 ± 0.01	NA	NA
*Culiseta annulata*	0.20 ± 0.01	0.16 ± 0.01	0.13 ± 0.01	0.11 ± 0.01
*Mansonia titillans*	0.06 ± 0.01	0.05 ± 0.01	NA	NA

## 4. Discussion

Our findings suggest that the introduction and establishment of non-native mosquitoes are driven, to some extent, by some ecological, life-history, and macroecological characteristics. Once established, vector mosquitoes can profoundly alter disease transmission dynamics, or introduce pathogens into previously unaffected regions [3]. Responding to their global invasion requires predictive tools that go beyond local surveillance and include proactive prevention and risk assessment. Our modelling approach achieved moderate to good predictive ability, stable across model repetitions, which indicates that the invasion potential of species can be inferred to some extent from trait data alone. Unlike previous efforts that modelled the potential distribution of species already introduced [60,61] our framework identifies species with high predicted invasion potential before they spread. Specifically, of the 169 species analyzed, 24 species with no prior invasion history received predicted introduction probabilities above the optimal TSS threshold, including 17 species that also had high predicted establishment probabilities.

A main result of our analysis is the consistent importance of native biogeographic origin in shaping predicted invasion potential. Native origin in Asia and Australia showed the highest relative importance for both introduction and establishment probabilities, whereas origin in Africa, the Americas, or Europe showed substantially lower importance and predicted probabilities. Native continent is a broad proxy that may capture several processes simultaneously, including historical trade connectivity, propagule pressure, environmental matching, and the prevalence of human-associated breeding habitats. This pattern is consistent with Asia’s recognized role as a major global source region for invasive species overall [62], including invasive insects [63] and recently introduced mosquitoes [5]. High human population density, intense containerized trade, rapid economic expansion, and the movement of commodities associated with mosquito breeding habitats have likely created substantial opportunities for mosquito transport from this region.

These biogeographic patterns likely reflect both species attributes and historical invasion opportunity. For example, following World War II, used aircraft tires were shipped from Asia and the Pacific region to the United States as part of postwar recovery and logistics operations, inadvertently facilitating the transport of mosquito eggs and larvae [64]. Today, globalization of plant and material trade continues to provide suitable introduction pathways. China remains the world’s largest bamboo exporter [65] and accounted for roughly 32% of global container port traffic in 2022 [66]. Large-scale initiatives such as the Belt and Road Initiative further expand trade connectivity across more than 120 countries [67], while the thawing of Arctic shipping routes may increasingly facilitate transport between Asia and Europe [68]. Thus, Asia’s importance in our models likely reflects both historical and ongoing source-region dynamics and may remain relevant for near-future surveillance if current trade patterns persist or intensify.

Australia’s importance mainly reflects the introduction of native species into the Pacific region and New Zealand, although several long-distance introductions have also been documented, including *Aedes vexans* in Hawaii [69], *Anopheles subpictus* in the Netherlands [70], and *Aedes notoscriptus* in California [71]. The latter illustrates how globalization and climatic similarity between native and introduced regions may jointly facilitate establishment success.

More broadly, native biogeographic origin likely integrates several dimensions relevant to invasion dynamics, including environmental matching and historical exposure to human-mediated transport pathways. Although models excluding biogeographic predictors produced broadly similar predictions, their consistently lower predictive performance (S2 Appendix) suggests that intrinsic species traits alone do not fully capture the processes shaping current mosquito invasions.

Concerning probability of species introduction, another main predictor identified is the use of human-made breeding sites. Species that oviposit in artificial habitats, plastic vessels, plant pots, rice fields, drainage systems or discarded tires, showed markedly higher probabilities of being transported and introduced than species restricted to natural water bodies. This finding aligns with classic invasion ecology, in which propagule pressure and human association are primary determinants of transport success [17,22,72]. Container breeding has the advantage that eggs and larvae persist in environments closely associated with human activity, trade and travel, from cargo holds to used-tire shipments [73]. This pattern, documented, *e.g.,* for *Ae. albopictus* and *Ae. japonicus* [11,73,74], is generalizable: nearly all species predicted with high probabilities of both introduction and establishment use human-made breeding sites. This underscores that invasion potential is closely coupled with the degree of adaptation to human-modified landscapes. Global urban expansion and the proliferation of disposable containers continue to multiply these breeding sites, providing abundant breeding opportunities for species with suitable ecological strategies [75,76].

Climatic predictors had a complementary influence, particularly on establishment success. The maximum annual precipitation of the native range was the strongest predictor of probability of species establishment, with probabilities rising sharply for species native from areas receiving approximately 1,500 mm yr⁻^1^. The relationship between minimum temperature and introduction probability was bimodal: species from both cold and warm native ranges were more likely to be introduced than those from mild climates. This dual pattern might just again stand as a proxy for the species native region, or it suggests that both cold-adapted and tropical mosquitoes possess distinct mechanisms enabling survival during transport, either tolerance to cold or resistance to dehydration. Species from warmer regions on the other hand were more likely to establish, with over half of the successfully established species originating from areas exceeding 34 °C in average maximum temperature. Again, the climate data used here reflect the conditions within the species’ native ranges.

Importantly, native-range climatic conditions considered here reflect where species occur, not necessarily the full range of conditions they may tolerate after introduction. This is well illustrated by *Ae. albopictus*, which was initially considered unlikely to establish far beyond its tropical and subtropical native range in Southeast Asia because it was assumed to require prolonged adaptation to novel environments and to be constrained by competition with local mosquito species [77]. Nevertheless, the species rapidly adapted to colder climates [78,79], outcompeted presumed competitors [80,81], and expanded across temperate regions worldwide. Accordingly, predictions of invasion and establishment potential based solely on native-range climatic conditions should be interpreted with caution.

We also found that widely distributed species were more likely to be introduced into new regions. This may translate into increased propagule pressure as greater distribution increases the chances of introduction [17]. This was however not observed for established species, which could indicate a filter for species establishment that is based more on intrinsic traits or adaptation to new environments [82,83].

Overall, our results indicate that mosquito invasion potential is best explained by the interaction between life-history flexibility, adaptation to human environments, and broad biogeographic exposure. Species associated with human-made breeding sites and originating from regions strongly connected to global trade networks showed higher probabilities of introduction. In turn, establishment probability was strongly associated with climatic conditions in the native range, particularly high precipitation regimes. Together, these results identify a characteristic profile of species with high predicted invasion potential: human-adapted species, often native to Asia and/or Australia, capable of exploiting human-made breeding sites and associated with climatic regimes characterized by high precipitation and thermal extremes. These findings extend previous qualitative insights [24] into a quantitative global framework, moving beyond the small number of well-studied medically important vectors (*e.g.,* [61,73,84,85]) and providing insight into the invasion potential of lesser-known species.

Beyond identifying traits of importance, our models identified 24 species with no recorded invasion history but trait profiles closely matching those of known invaders. Of these, 17 species were also predicted with high probabilities of establishment, making them species with high predicted invasion potential, and identifying them as priority candidates for early surveillance. For example, *Culex vishnui,* the species with the highest predicted introduction probability and likely to establish is native to South and East Asia, where it thrives in rice fields, ground pools, and small artificial containers [21]. It has been found naturally infected with multiple arboviruses, including Japanese encephalitis and West Nile virus [86,87]. *Anopheles culicifacies*, ranking second in our high risk species list, is an important malaria vector, particularly in South and Southwest Asia. [88]. Predominantly anthropophilic but occasionally zoophilic, it can take multiple blood meals per gonotrophic cycle [21] increasing its potential for pathogen transmission. *Aedes lineatopennis*, native to the Australasian and Oriental regions, was first described from the Philippines [21,89]. It has been found naturally infected with Japanese encephalitis [90] and Middelburg virus [91], and is considered a potential vector for Ross River and Murray Valley encephalitis viruses [92,93].

Our results provide valuable insights, but their interpretation should also take certain limitations into account. First, trait data for some mosquitoes remains partly incomplete. Similar to other studies [16,17,94], we used a trait imputation approach to overcome this limitation. To minimize bias, we included only species with at least 75% trait completeness, and only variables that were known for at least 75% of the species. The standard deviations of our predictions were low, indicating that model outputs were not highly sensitive to the imputation process. Nonetheless, imputation may obscure subtle ecological differences. Second, because our approach is species trait–centric, it does not account for the local conditions species encounter at introduction sites, such as climate, biotic interactions, or vector control measures that can influence invasion outcomes [95,96]. Third, the delineation of native origin follows a broad continental-level classification that ignores differences between subregions within individual continents, which may differentially influence invasion potential. Fourth, several predictors used here are not static. Global trade networks, transport routes, and commodity flows may change, and regions that are currently less important sources may become more important in the future. Thus, although our results provide a robust baseline for identifying species with elevated invasion potential under present-day ecological and trade conditions, they should be complemented by geographically explicit establishment-risk assessments and updated pathway information.

## 5. Conclusion

Overall, our results indicate that the invasion potential of mosquitoes can be partially predicted from measurable ecological, life-history, and macroecological traits. Species associated with human-made breeding sites, broad native distributions, regions of high precipitation, and historically important source regions showed the highest predicted probabilities of introduction and establishment. By identifying these species and their characteristic profiles, our approach underscores the potential for proactive, trait-based surveillance strategies that extend beyond the currently recognized vector species. Trait-based frameworks such as the one presented here can support early-warning systems, guide allocation of surveillance resources, and ultimately reduce the risk of novel vector-borne disease emergence. Once invasive mosquitoes succeed in establishing populations, their control and eradication become exceedingly challenging [97,98]. In this context, preventing introductions remains the most efficient and economically viable strategy.

## Supporting information

S1 Appendix Traits data set. Data set generated with all species traits combinations and corresponding sources of information.
(XLSX)

S2 Appendix Supplementary material. Supplementary information and model results of models using only ecological and life-history traits, without biogeographic traits.
(PDF)

S3 Appendix R code for analysis. Compressed folder containing the R script used to perform the analyses, along with a simplified version of the input dataset. The full code repository is also available on GitHub: https://github.com/rebe3000/mosquitoinvasionpotential. The original global mosquito introduction dataset can be accessed at: https://doi.org/10.5281/zenodo.15731141.
(ZIP)
