# Supplementary material for: Trait-based prediction of disease-vector mosquito invasion potential
Source: PLoS Negl Trop Dis. 2026 Jul 23;20(7):e0014538. doi: 10.1371/journal.pntd.0014538 (PMC13395358; doi:10.1371/journal.pntd.0014538)
Supplement: S2 Appendix — Supplementary information and model results of models using only ecological and life-history traits, without biogeographic traits. (PDF) [file pntd.0014538.s002.pdf]

S2 Appendix for:

Trait based assessment of the invasion potential of disease vector mosquitoes

Authors

Rebecca Pabst[1*], Carla A. Sousa[1], César Capinha[2,3*]

Affiliations

[1] Global Health and Tropical Medicine, GHTM, LA-REAL, Institute of Hygiene and Tropical Medicine, IHMT, NOVA University Lisbon, Lisbon, Portugal.

[2] Centre of Geographical Studies, Institute of Geography and Spatial Planning, University of Lisbon, Lisboa, Portugal.

[3] Associate Laboratory TERRA, Lisboa, Portugal.

* Corresponding authors

E-mails: pabst.rebecca@gmail.com; cesarcapinha@edu.ulisboa.pt

5 Figures

6 Tables

Table A. Mosquito species included in the models, their observed invasion-status variables, and ensemble model predictions. The table includes the 169 species with sufficient trait data and occurrence information for modelling. Introduced indicates at least one confirmed record outside the native range; Introduced post-1950 indicates at least one such record after 1950. Established indicates confirmed self-sustaining populations outside the native range, considering either all records or only records after 1950, as specified by the column. All variables are binary: 0 = no, 1 = yes. Ensemble model predictions from the full models in the main article are shown as mean predicted probabilities ± SD across 100 model repetitions for each response variable.

Species	introduced	introduced post-1950	established	established post-1950	predicted introduction probability	predicted introduction post-1950 probability	predicted establishment probability	predicted establishment post-1950 probability
Aedeomyia squamipennis	0	0	0	0	0.16 ± 0.01	0.15 ± 0.01	0.07 ± 0.01	0.06 ± 0.01
Aedes aegypti	1	1	1	1	0.41 ± 0.01	0.33 ± 0.01	0.24 ± 0.01	0.24 ± 0.01
Aedes africanus	0	0	0	0	0.29 ± 0.01	0.28 ± 0.01	0.29 ± 0.01	0.28 ± 0.01
Aedes albifasciatus	0	0	0	0	0.26 ± 0.01	0.23 ± 0.01	0.13 ± 0.01	0.11 ± 0.01
Aedes albopictus	1	1	1	1	0.65 ± 0.01	0.51 ± 0.01	0.61 ± 0.01	0.42 ± 0.02
Aedes apicoargenteus	0	0	0	0	0.09 ± 0.01	0.08 ± 0.01	0.04 ± 0.01	0.04 ± 0.01
Aedes argenteopunctatus	0	0	0	0	0.20 ± 0.01	0.23 ± 0.01	0.17 ± 0.01	0.16 ± 0.01
Aedes atlanticus	0	0	0	0	0.19 ± 0.01	0.20 ± 0.01	0.06 ± 0.01	0.05 ± 0.01
Aedes atropalpus	1	1	1	1	0.32 ± 0.01	0.31 ± 0.01	0.11 ± 0.01	0.10 ± 0.01
Aedes caballus	0	0	0	0	0.25 ± 0.01	0.22 ± 0.01	0.13 ± 0.01	0.11 ± 0.01
Aedes camptorhynchus	1	1	0	0	0.33 ± 0.01	0.31 ± 0.01	0.21 ± 0.01	0.20 ± 0.01
Aedes canadensis	0	0	0	0	0.18 ± 0.01	0.20 ± 0.01	0.08 ± 0.01	0.09 ± 0.01
Aedes cantans	0	0	0	0	0.04 ± 0.01	0.03 ± 0.00	0.02 ± 0.00	0.02 ± 0.00
Aedes cantator	0	0	0	0	0.20 ± 0.01	0.20 ± 0.01	0.03 ± 0.01	0.03 ± 0.00
Aedes caspius	0	0	0	0	0.18 ± 0.01	0.19 ± 0.01	0.15 ± 0.01	0.14 ± 0.01
Aedes cataphylla	0	0	0	0	0.05 ± 0.01	0.04 ± 0.01	0.02 ± 0.00	0.02 ± 0.00
Aedes cinereus	0	0	0	0	0.08 ± 0.01	0.06 ± 0.01	0.03 ± 0.00	0.03 ± 0.00
Aedes circumluteolus	0	0	0	0	0.05 ± 0.01	0.05 ± 0.01	0.01 ± 0.00	0.02 ± 0.00
Aedes communis	0	0	0	0	0.06 ± 0.01	0.06 ± 0.01	0.02 ± 0.00	0.03 ± 0.00
Aedes cumminsii	0	0	0	0	0.13 ± 0.01	0.13 ± 0.01	0.02 ± 0.00	0.02 ± 0.00
Aedes dalzieli	0	0	0	0	0.08 ± 0.01	0.09 ± 0.01	0.06 ± 0.01	0.06 ± 0.01
Aedes dentatus	0	0	0	0	0.11 ± 0.01	0.09 ± 0.01	0.03 ± 0.00	0.03 ± 0.00
Aedes detritus	0	0	0	0	0.27 ± 0.01	0.18 ± 0.01	0.15 ± 0.01	0.12 ± 0.01

Aedes diantaeus	0	0	0	0	0.11 ± 0.01	0.10 ± 0.01	0.08 ± 0.01	0.09 ± 0.01
Aedes dorsalis	0	0	0	0	0.10 ± 0.01	0.08 ± 0.01	0.07 ± 0.01	0.07 ± 0.01
Aedes excrucians	0	0	0	0	0.02 ± 0.00	0.01 ± 0.00	0.01 ± 0.00	0.01 ± 0.00
Aedes fulvus	0	0	0	0	0.14 ± 0.01	0.13 ± 0.01	0.07 ± 0.01	0.06 ± 0.01
Aedes furcifer	0	0	0	0	0.10 ± 0.01	0.11 ± 0.01	0.07 ± 0.01	0.07 ± 0.01
Aedes geniculatus	0	0	0	0	0.40 ± 0.01	0.29 ± 0.01	0.31 ± 0.01	0.27 ± 0.01
Aedes hexodontus	0	0	0	0	0.10 ± 0.01	0.09 ± 0.01	0.10 ± 0.01	0.10 ± 0.01
Aedes hirsutus	0	0	0	0	0.15 ± 0.01	0.12 ± 0.01	0.08 ± 0.01	0.09 ± 0.01
Aedes infirmatus	0	0	0	0	0.15 ± 0.01	0.15 ± 0.01	0.04 ± 0.01	0.04 ± 0.01
Aedes japonicus	1	1	1	1	0.50 ± 0.01	0.24 ± 0.01	0.29 ± 0.01	0.17 ± 0.01
Aedes lineatopennis	0	0	0	0	0.57 ± 0.01	0.52 ± 0.01	0.39 ± 0.01	0.36 ± 0.01
Aedes luteocephalus	0	0	0	0	0.14 ± 0.01	0.14 ± 0.01	0.12 ± 0.01	0.14 ± 0.01
Aedes mcintoshi	1	1	0	0	0.05 ± 0.01	0.04 ± 0.01	0.02 ± 0.00	0.03 ± 0.00
Aedes melanimon	0	0	0	0	0.23 ± 0.01	0.21 ± 0.01	0.11 ± 0.01	0.12 ± 0.01
Aedes metallicus	0	0	0	0	0.10 ± 0.01	0.09 ± 0.01	0.07 ± 0.01	0.07 ± 0.01
Aedes mitchellae	0	0	0	0	0.09 ± 0.01	0.09 ± 0.01	0.01 ± 0.00	0.01 ± 0.00
Aedes notoscriptus	1	1	1	1	0.50 ± 0.02	0.38 ± 0.02	0.25 ± 0.01	0.21 ± 0.01
Aedes ochraceus	0	0	0	0	0.22 ± 0.01	0.20 ± 0.01	0.02 ± 0.00	0.02 ± 0.00
Aedes pembaensis	0	0	0	0	0.23 ± 0.01	0.23 ± 0.01	0.10 ± 0.01	0.10 ± 0.01
Aedes polynesiensis	1	1	0	0	0.43 ± 0.02	0.37 ± 0.01	0.25 ± 0.01	0.22 ± 0.01
Aedes punctor	0	0	0	0	0.05 ± 0.01	0.06 ± 0.01	0.06 ± 0.01	0.07 ± 0.01
Aedes scapularis	0	0	0	0	0.27 ± 0.01	0.24 ± 0.01	0.20 ± 0.01	0.19 ± 0.01
Aedes scutellaris	1	0	0	0	0.67 ± 0.01	0.66 ± 0.01	0.48 ± 0.02	0.43 ± 0.01
Aedes serratus	0	0	0	0	0.06 ± 0.01	0.06 ± 0.01	0.07 ± 0.01	0.07 ± 0.01
Aedes sexlineatus	0	0	0	0	0.11 ± 0.01	0.13 ± 0.01	0.09 ± 0.01	0.10 ± 0.01
Aedes sollicitans	0	0	0	0	0.22 ± 0.01	0.20 ± 0.01	0.08 ± 0.01	0.08 ± 0.01
Aedes sticticus	0	0	0	0	0.03 ± 0.01	0.03 ± 0.00	0.03 ± 0.00	0.03 ± 0.00
Aedes stimulans	0	0	0	0	0.10 ± 0.01	0.10 ± 0.01	0.04 ± 0.00	0.04 ± 0.01
Aedes taeniorhynchus	0	0	0	0	0.28 ± 0.01	0.23 ± 0.01	0.21 ± 0.01	0.17 ± 0.01
Aedes taylori	0	0	0	0	0.17 ± 0.01	0.17 ± 0.01	0.12 ± 0.01	0.10 ± 0.01
Aedes togoi	1	0	1	0	0.64 ± 0.02	0.61 ± 0.01	0.54 ± 0.02	0.51 ± 0.01
Aedes triseriatus	1	1	0	0	0.25 ± 0.01	0.27 ± 0.01	0.25 ± 0.01	0.24 ± 0.01
Aedes trivittatus	0	0	0	0	0.18 ± 0.01	0.16 ± 0.01	0.03 ± 0.00	0.03 ± 0.01
Aedes unilineatus	1	1	1	1	0.35 ± 0.01	0.33 ± 0.02	0.33 ± 0.02	0.30 ± 0.01

Aedes vexans	1	1	1	1	0.38 ± 0.01	0.35 ± 0.01	0.20 ± 0.01	0.21 ± 0.01
Aedes vigilax	1	1	1	1	0.49 ± 0.01	0.45 ± 0.01	0.17 ± 0.01	0.17 ± 0.01
Aedes vittatus	1	1	1	1	0.46 ± 0.01	0.38 ± 0.01	0.37 ± 0.01	0.34 ± 0.01
Anopheles albimanus	1	1	1	1	0.42 ± 0.01	0.36 ± 0.02	0.32 ± 0.01	0.26 ± 0.01
Anopheles albitarsis	0	0	0	0	0.21 ± 0.01	0.14 ± 0.01	0.09 ± 0.01	0.09 ± 0.01
Anopheles amictus	0	0	0	0	0.56 ± 0.01	0.52 ± 0.01	0.26 ± 0.01	0.26 ± 0.01
Anopheles aquasalis	0	0	0	0	0.22 ± 0.01	0.22 ± 0.01	0.16 ± 0.01	0.15 ± 0.01
Anopheles arabiensis	1	1	1	0	0.65 ± 0.01	0.60 ± 0.01	0.40 ± 0.01	0.41 ± 0.01
Anopheles atroparvus	0	0	0	0	0.27 ± 0.01	0.17 ± 0.01	0.15 ± 0.01	0.14 ± 0.01
Anopheles bancroftii	1	1	1	1	0.38 ± 0.02	0.35 ± 0.01	0.18 ± 0.01	0.18 ± 0.01
Anopheles barbirostris	1	1	0	0	0.63 ± 0.01	0.47 ± 0.02	0.48 ± 0.01	0.42 ± 0.01
Anopheles bellator	0	0	0	0	0.04 ± 0.01	0.05 ± 0.01	0.02 ± 0.00	0.02 ± 0.00
Anopheles braziliensis	0	0	0	0	0.04 ± 0.01	0.04 ± 0.01	0.02 ± 0.00	0.02 ± 0.00
Anopheles brunnipes	0	0	0	0	0.11 ± 0.01	0.07 ± 0.01	0.04 ± 0.01	0.04 ± 0.01
Anopheles carnevalei	0	0	0	0	0.10 ± 0.01	0.11 ± 0.01	0.02 ± 0.00	0.02 ± 0.00
Anopheles cinereus	0	0	0	0	0.21 ± 0.01	0.16 ± 0.01	0.06 ± 0.01	0.06 ± 0.01
Anopheles claviger	0	0	0	0	0.42 ± 0.01	0.29 ± 0.01	0.25 ± 0.01	0.24 ± 0.01
Anopheles coustani	1	0	0	0	0.46 ± 0.01	0.41 ± 0.01	0.17 ± 0.01	0.15 ± 0.01
Anopheles crucians	1	1	0	0	0.06 ± 0.01	0.05 ± 0.01	0.02 ± 0.00	0.02 ± 0.00
Anopheles cruzii	0	0	0	0	0.08 ± 0.01	0.09 ± 0.01	0.06 ± 0.01	0.06 ± 0.01
Anopheles culicifacies	0	0	0	0	0.70 ± 0.01	0.56 ± 0.01	0.41 ± 0.01	0.42 ± 0.01
Anopheles darlingi	1	1	1	1	0.11 ± 0.01	0.10 ± 0.01	0.08 ± 0.01	0.06 ± 0.01
Anopheles fluviatilis	0	0	0	0	0.40 ± 0.01	0.31 ± 0.01	0.09 ± 0.01	0.09 ± 0.01
Anopheles funestus	1	0	0	0	0.38 ± 0.01	0.39 ± 0.01	0.32 ± 0.01	0.23 ± 0.01
Anopheles gambiae s.s.	1	1	1	1	0.65 ± 0.01	0.41 ± 0.01	0.30 ± 0.01	0.18 ± 0.01
Anopheles grabhamii	0	0	0	0	0.27 ± 0.01	0.25 ± 0.01	0.11 ± 0.01	0.11 ± 0.01
Anopheles hancocki	0	0	0	0	0.07 ± 0.01	0.06 ± 0.01	0.03 ± 0.00	0.04 ± 0.01
Anopheles hyrcanus	0	0	0	0	0.45 ± 0.01	0.37 ± 0.02	0.34 ± 0.01	0.28 ± 0.01
Anopheles labranchiae	0	0	0	0	0.18 ± 0.01	0.17 ± 0.01	0.12 ± 0.01	0.11 ± 0.01
Anopheles litoralis	1	1	0	0	0.33 ± 0.01	0.26 ± 0.02	0.24 ± 0.01	0.18 ± 0.01
Anopheles maculipalpis	0	0	0	0	0.17 ± 0.01	0.10 ± 0.01	0.02 ± 0.00	0.02 ± 0.00

Anopheles maculipennis	1	0	0	0	0.20 ± 0.01	0.18 ± 0.01	0.15 ± 0.01	0.14 ± 0.01
Anopheles mediopunctatus	0	0	0	0	0.03 ± 0.01	0.03 ± 0.00	0.02 ± 0.00	0.02 ± 0.00
Anopheles melas	0	0	0	0	0.19 ± 0.01	0.17 ± 0.01	0.11 ± 0.01	0.10 ± 0.01
Anopheles merus	0	0	0	0	0.23 ± 0.01	0.20 ± 0.02	0.06 ± 0.01	0.06 ± 0.01
Anopheles moucheti	0	0	0	0	0.10 ± 0.01	0.08 ± 0.01	0.03 ± 0.01	0.04 ± 0.01
Anopheles neivai	0	0	0	0	0.08 ± 0.01	0.09 ± 0.01	0.08 ± 0.01	0.09 ± 0.01
Anopheles neomaculipalpus	0	0	0	0	0.09 ± 0.01	0.08 ± 0.01	0.04 ± 0.00	0.04 ± 0.01
Anopheles nili	0	0	0	0	0.23 ± 0.01	0.13 ± 0.01	0.02 ± 0.00	0.03 ± 0.00
Anopheles nuneztovari	0	0	0	0	0.26 ± 0.01	0.28 ± 0.01	0.23 ± 0.01	0.24 ± 0.01
Anopheles oswaldoi	0	0	0	0	0.14 ± 0.01	0.13 ± 0.01	0.12 ± 0.01	0.13 ± 0.01
Anopheles pharoensis	1	1	0	0	0.27 ± 0.01	0.13 ± 0.01	0.08 ± 0.01	0.05 ± 0.01
Anopheles plumbeus	0	0	0	0	0.42 ± 0.01	0.32 ± 0.01	0.33 ± 0.01	0.29 ± 0.01
Anopheles pseudopunctipennis	0	0	0	0	0.37 ± 0.01	0.31 ± 0.01	0.17 ± 0.01	0.20 ± 0.01
Anopheles pulcherrimus	0	0	0	0	0.31 ± 0.01	0.30 ± 0.01	0.13 ± 0.01	0.12 ± 0.01
Anopheles punctimacula	0	0	0	0	0.11 ± 0.01	0.11 ± 0.01	0.09 ± 0.01	0.08 ± 0.01
Anopheles punctipennis	1	1	0	0	0.20 ± 0.01	0.19 ± 0.01	0.09 ± 0.01	0.09 ± 0.01
Anopheles quadrimaculatus	0	0	0	0	0.31 ± 0.01	0.21 ± 0.01	0.16 ± 0.01	0.11 ± 0.01
Anopheles rufipes	0	0	0	0	0.43 ± 0.02	0.31 ± 0.01	0.12 ± 0.01	0.10 ± 0.01
Anopheles sacharovi	0	0	0	0	0.29 ± 0.01	0.16 ± 0.01	0.17 ± 0.01	0.15 ± 0.01
Anopheles sergentii	0	0	0	0	0.32 ± 0.01	0.30 ± 0.01	0.15 ± 0.01	0.12 ± 0.01
Anopheles stephensi	1	1	1	1	0.60 ± 0.01	0.64 ± 0.01	0.58 ± 0.01	0.49 ± 0.02
Anopheles subpictus	1	1	1	1	0.71 ± 0.01	0.67 ± 0.01	0.54 ± 0.01	0.47 ± 0.01
Anopheles superpictus	0	0	0	0	0.24 ± 0.01	0.18 ± 0.01	0.16 ± 0.01	0.12 ± 0.01
Anopheles triannulatus	0	0	0	0	0.20 ± 0.01	0.19 ± 0.01	0.15 ± 0.01	0.12 ± 0.01
Anopheles walkeri	0	0	0	0	0.08 ± 0.01	0.07 ± 0.01	0.03 ± 0.00	0.02 ± 0.00
Armigeres subalbatus	1	1	1	1	0.66 ± 0.01	0.59 ± 0.01	0.63 ± 0.01	0.57 ± 0.01
Coquillettidia linealis	0	0	0	0	0.36 ± 0.01	0.36 ± 0.01	0.10 ± 0.01	0.10 ± 0.01
Coquillettidia perturbans	0	0	0	0	0.10 ± 0.01	0.11 ± 0.01	0.02 ± 0.00	0.03 ± 0.00
Coquillettidia richiardii	0	0	0	0	0.32 ± 0.01	0.21 ± 0.01	0.14 ± 0.01	0.14 ± 0.01
Coquillettidia venezuelensis	0	0	0	0	0.04 ± 0.01	0.04 ± 0.01	0.02 ± 0.00	0.02 ± 0.00

Culex annulirostris	1	1	0	0	0.55 ± 0.01	0.55 ± 0.02	0.43 ± 0.02	0.42 ± 0.01
Culex antennatus	1	1	0	0	0.46 ± 0.01	0.44 ± 0.01	0.41 ± 0.01	0.26 ± 0.01
Culex bitaeniorhynchus	1	1	0	0	0.67 ± 0.01	0.68 ± 0.01	0.67 ± 0.01	0.59 ± 0.01
Culex coronator	1	1	1	1	0.11 ± 0.01	0.13 ± 0.01	0.07 ± 0.01	0.09 ± 0.01
Culex erraticus	0	0	0	0	0.18 ± 0.01	0.19 ± 0.01	0.09 ± 0.01	0.10 ± 0.01
Culex fuscocephala	1	1	1	1	0.64 ± 0.01	0.63 ± 0.01	0.57 ± 0.01	0.53 ± 0.01
Culex gelidus	1	1	1	1	0.62 ± 0.01	0.57 ± 0.01	0.41 ± 0.01	0.38 ± 0.01
Culex modestus	1	1	1	1	0.33 ± 0.01	0.25 ± 0.01	0.20 ± 0.01	0.18 ± 0.01
Culex nigripalpus	0	0	0	0	0.42 ± 0.02	0.42 ± 0.01	0.33 ± 0.01	0.26 ± 0.01
Culex ocossa	0	0	0	0	0.09 ± 0.01	0.08 ± 0.01	0.06 ± 0.01	0.07 ± 0.01
Culex perexiguus	0	0	0	0	0.45 ± 0.02	0.46 ± 0.02	0.27 ± 0.01	0.25 ± 0.01
Culex pipiens s.s.	1	1	1	1	0.43 ± 0.01	0.32 ± 0.01	0.30 ± 0.01	0.27 ± 0.01
Culex poicilipes	0	0	0	0	0.45 ± 0.01	0.38 ± 0.02	0.24 ± 0.01	0.22 ± 0.01
Culex portesi	0	0	0	0	0.05 ± 0.01	0.06 ± 0.01	0.05 ± 0.01	0.06 ± 0.01
Culex quinquefasciatus	1	1	1	1	0.67 ± 0.01	0.68 ± 0.01	0.64 ± 0.01	0.51 ± 0.01
Culex restuans	0	0	0	0	0.28 ± 0.01	0.29 ± 0.01	0.16 ± 0.01	0.16 ± 0.01
Culex rubinotus	0	0	0	0	0.10 ± 0.01	0.10 ± 0.01	0.08 ± 0.01	0.07 ± 0.01
Culex salinarius	0	0	0	0	0.26 ± 0.01	0.27 ± 0.02	0.09 ± 0.01	0.09 ± 0.01
Culex sitiens	1	1	1	0	0.73 ± 0.01	0.70 ± 0.01	0.56 ± 0.01	0.51 ± 0.01
Culex spissipes	0	0	0	0	0.04 ± 0.01	0.04 ± 0.01	0.04 ± 0.01	0.04 ± 0.01
Culex taeniopus	0	0	0	0	0.15 ± 0.01	0.14 ± 0.01	0.07 ± 0.01	0.07 ± 0.01
Culex tarsalis	1	1	1	1	0.29 ± 0.01	0.25 ± 0.01	0.25 ± 0.01	0.20 ± 0.01
Culex theileri	0	0	0	0	0.55 ± 0.01	0.50 ± 0.01	0.41 ± 0.02	0.37 ± 0.01
Culex torrentium	0	0	0	0	0.46 ± 0.01	0.44 ± 0.02	0.37 ± 0.02	0.36 ± 0.01
Culex tritaeniorhynchus	1	1	1	1	0.71 ± 0.01	0.71 ± 0.01	0.53 ± 0.01	0.50 ± 0.01
Culex univittatus	0	0	0	0	0.57 ± 0.02	0.54 ± 0.01	0.44 ± 0.01	0.38 ± 0.01
Culex vishnui	0	0	0	0	0.89 ± 0.01	0.85 ± 0.01	0.58 ± 0.01	0.51 ± 0.01
Culex vomerifer	0	0	0	0	0.03 ± 0.00	0.04 ± 0.00	0.03 ± 0.00	0.03 ± 0.00
Culiseta annulata	1	1	1	1	0.20 ± 0.01	0.16 ± 0.01	0.13 ± 0.01	0.11 ± 0.01
Culiseta inornata	0	0	0	0	0.40 ± 0.01	0.42 ± 0.01	0.34 ± 0.01	0.33 ± 0.01
Culiseta melanura	0	0	0	0	0.07 ± 0.01	0.08 ± 0.01	0.03 ± 0.00	0.04 ± 0.01
Culiseta morsitans	0	0	0	0	0.23 ± 0.01	0.23 ± 0.01	0.24 ± 0.01	0.24 ± 0.01
Eretmapodites chrysogaster	0	0	0	0	0.08 ± 0.01	0.08 ± 0.01	0.06 ± 0.01	0.06 ± 0.01

Haemagogus leucocelaenus	0	0	0	0	0.10 ± 0.01	0.11 ± 0.01	0.06 ± 0.01	0.06 ± 0.01
Haemagogus mesodentatus	0	0	0	0	0.18 ± 0.01	0.19 ± 0.01	0.15 ± 0.01	0.12 ± 0.01
Haemagogus spegazzinii	0	0	0	0	0.09 ± 0.01	0.10 ± 0.01	0.08 ± 0.01	0.09 ± 0.01
Limatus asulleptus	0	0	0	0	0.17 ± 0.01	0.15 ± 0.01	0.10 ± 0.01	0.07 ± 0.01
Mansonia africana	0	0	0	0	0.25 ± 0.01	0.25 ± 0.01	0.07 ± 0.01	0.09 ± 0.01
Mansonia septempunctata	0	0	0	0	0.50 ± 0.01	0.51 ± 0.01	0.32 ± 0.01	0.31 ± 0.01
Mansonia titillans	1	1	0	0	0.06 ± 0.01	0.05 ± 0.00	0.05 ± 0.01	0.05 ± 0.01
Mansonia uniformis	1	1	1	1	0.69 ± 0.01	0.60 ± 0.01	0.42 ± 0.01	0.39 ± 0.01
Psorophora albigenu	0	0	0	0	0.09 ± 0.01	0.11 ± 0.01	0.08 ± 0.01	0.08 ± 0.01
Psorophora ciliata	0	0	0	0	0.26 ± 0.01	0.24 ± 0.01	0.16 ± 0.01	0.16 ± 0.01
Psorophora columbiae	0	0	0	0	0.20 ± 0.01	0.19 ± 0.01	0.09 ± 0.01	0.09 ± 0.01
Psorophora confinnis	0	0	0	0	0.26 ± 0.01	0.29 ± 0.01	0.29 ± 0.01	0.30 ± 0.01
Psorophora ferox	0	0	0	0	0.17 ± 0.01	0.18 ± 0.01	0.04 ± 0.00	0.04 ± 0.01
Sabethes chloropterus	0	0	0	0	0.11 ± 0.01	0.11 ± 0.01	0.09 ± 0.01	0.08 ± 0.01
Trichoprosopon digitatum	0	0	0	0	0.09 ± 0.01	0.10 ± 0.01	0.08 ± 0.01	0.08 ± 0.01
Uranotaenia sapphirina	0	0	0	0	0.11 ± 0.01	0.11 ± 0.01	0.03 ± 0.01	0.04 ± 0.00
Wyeomyia melanocephala	0	0	0	0	0.05 ± 0.01	0.05 ± 0.01	0.04 ± 0.01	0.03 ± 0.00
Wyeomyia occulta	0	0	0	0	0.10 ± 0.01	0.08 ± 0.01	0.06 ± 0.01	0.05 ± 0.01

Table B. Species traits compiled for the 184 mosquito species of medical importance. Traits include ecological, life-history, and macroecological predictors used to characterize species' introduction and establishment potential.

**Trait**	**Definition**	**Information recorded in variable**
Oviposition in non-human-made breeding sites	Whether a species lays eggs in natural meaning non-human-made breeding sites. Examples include tree holes, swamps, rivers.	Classes: yes / no
Oviposition in human-made breeding sites	Whether a species lays eggs in human-made breeding sites. Examples include plastic container, vase, man-made cut bamboo that collects water, drainage ditch, rice field.	Classes: yes / no
Oviposition in small breeding sites	Whether a species lays eggs in breeding sites smaller than 1 m^2. Examples include tree holes, coconut shells, plastic containers, rockpools.	Classes: yes / no

Oviposition in large breeding sites	Whether a species lays eggs in breeding sites larger than 1 m^2. Examples include swamps, river margins, swimming pools.	Classes: yes / no
Fresh water	Whether a species can develop in fresh water.	Classes: yes / no
Brackish water	Whether a species can develop in brackish water.	Classes: yes / no
Salt water	Whether a species can develop in salt water.	Classes: yes / no
Egg survives without water	The ability of eggs to survive without water for long periods of time. Short-time means that eggs from dry soil samples or under experimental conditions could still be incubated in some cases after up to 14 days.	Classes: yes / no / short-time
Desiccation resistance	Ability of eggs to resist desiccation (i.e. survive after drying).	Classes: yes / no
Oviposition strategy	The way in which a species lays its eggs.	Classes: In rafts / individually / individually but clustered / clustered underwater
Minimum mean annual temperatures of its native range	Minimum mean annual minimum temperature at which the species was observed. The extracted value corresponds to the 97.5% percentile of mean annual minimum temperatures at observation sites within countries where the species occurred naturally (in its native range).	Continuous value (°C)
Maximum mean annual temperatures of its native range	Maximum mean annual maximum temperature at which the species was observed. The extracted value corresponds to the 97.5% percentile of mean annual maximum temperature at observation sites within countries where the species occurred naturally (in its native range).	Continuous value (°C)
Minimum sum of annual precipitation of its native range	Minimum sum of annual precipitation at which the species was observed. The extracted value corresponds to the 97.5% percentile of minimum sum of annual precipitation at observation sites within countries where the species occurred naturally (in its native range).	Continuous value (mm)
Maximum sum of annual precipitation of its native range	Maximum sum of annual precipitation at which the species was observed. The extracted value corresponds to the 97.5% percentile of maximum sum of annual precipitation at observation sites within countries where the species occurred naturally (in its native range).	Continuous value (mm)
Native distribution range (whole country area)	Distribution range of species based on the total area of countries in which the species occurred in its native range.	Continuous value (km^2)
Native to Africa	Whether a species is native to the African continent.	Classes: yes / no
Native to Asia	Whether a species is native to the Asian continent.	Classes: yes / no
Native to Australia	Whether a species is native to the Australian continent.	Classes: yes / no
Native to Europe	Whether a species is native to the European continent.	Classes: yes / no
Native to North America	Whether a species is native to the North American continent.	Classes: yes / no
Native to South America	Whether a species is native to the South American continent.	Classes: yes / no

Proportion amphibian blood meal host	Proportion of bloodmeals derived from amphibian hosts. Together, the amphibian, avian, mammalian, and reptilian values sum to 1.	Proportion, between 0 and 1.
Proportion avian blood meal host	Proportion of bloodmeals derived from avian hosts. Together, the amphibian, avian, mammalian, and reptilian values sum to 1.	Proportion, between 0 and 1.
Proportion mammalian blood meal host	Proportion of bloodmeals derived from mammalian hosts. Together, the amphibian, avian, mammalian, and reptilian values sum to 1.	Proportion, between 0 and 1.
Proportion reptilian blood meal host	Proportion of bloodmeals derived from reptilian hosts. Together, the amphibian, avian, mammalian, and reptilian values sum to 1.	Proportion, between 0 and 1.

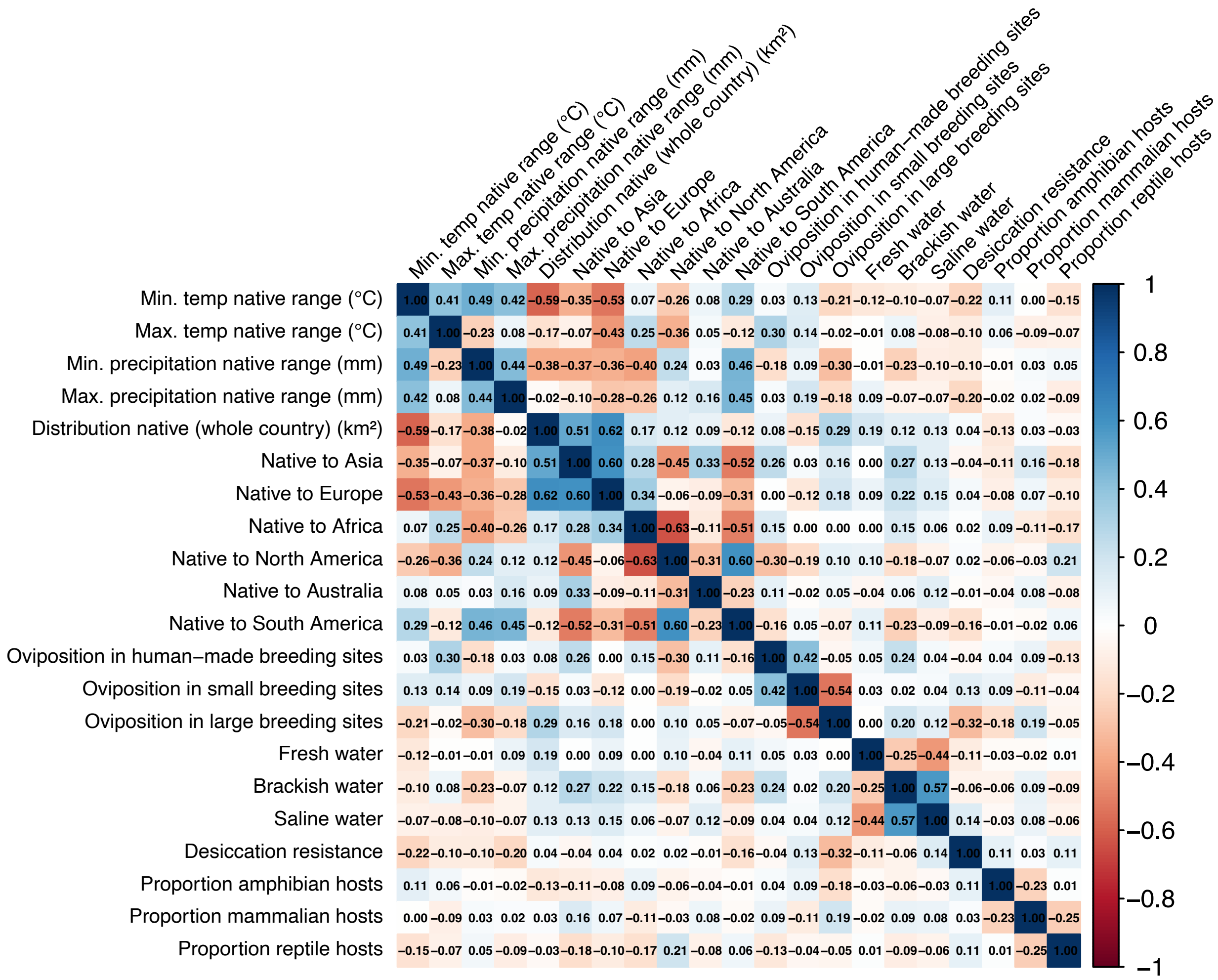

Figure A. Pairwise Pearson correlation matrix of numerical predictor variables retained after correlation filtering. Only variables kept for model fitting are shown.

Table C. LOOCV random forest model performance for models with the full set of traits presented in the main manuscript. Values show mean ± SD across 100 model repetitions for models predicting introduction, introduction after 1950, establishment, and establishment after 1950.

Response	AUC	OOB error	Threshold tss	Accuracy	Sensitivity	Specificity	Precision	F1
Introduced	0.82±0	0.14±0	0.30±0.01	0.80±0.01	0.78±0.02	0.80±0.02	0.60±0.02	0.65±0.01
Introduced after 1950	0.78±0	0.15±0	0.26±0.03	0.76±0.03	0.76±0.06	0.76±0.05	0.51±0.04	0.58±0.01
Established	0.85±0	0.11±0	0.17±0.01	0.77±0.01	0.85±0.02	0.75±0.02	0.42±0.01	0.56±0.01
Established after 1950	0.81±0	0.11±0	0.16±0.01	0.74±0.01	0.84±0.01	0.73±0.02	0.36±0.01	0.50±0.01

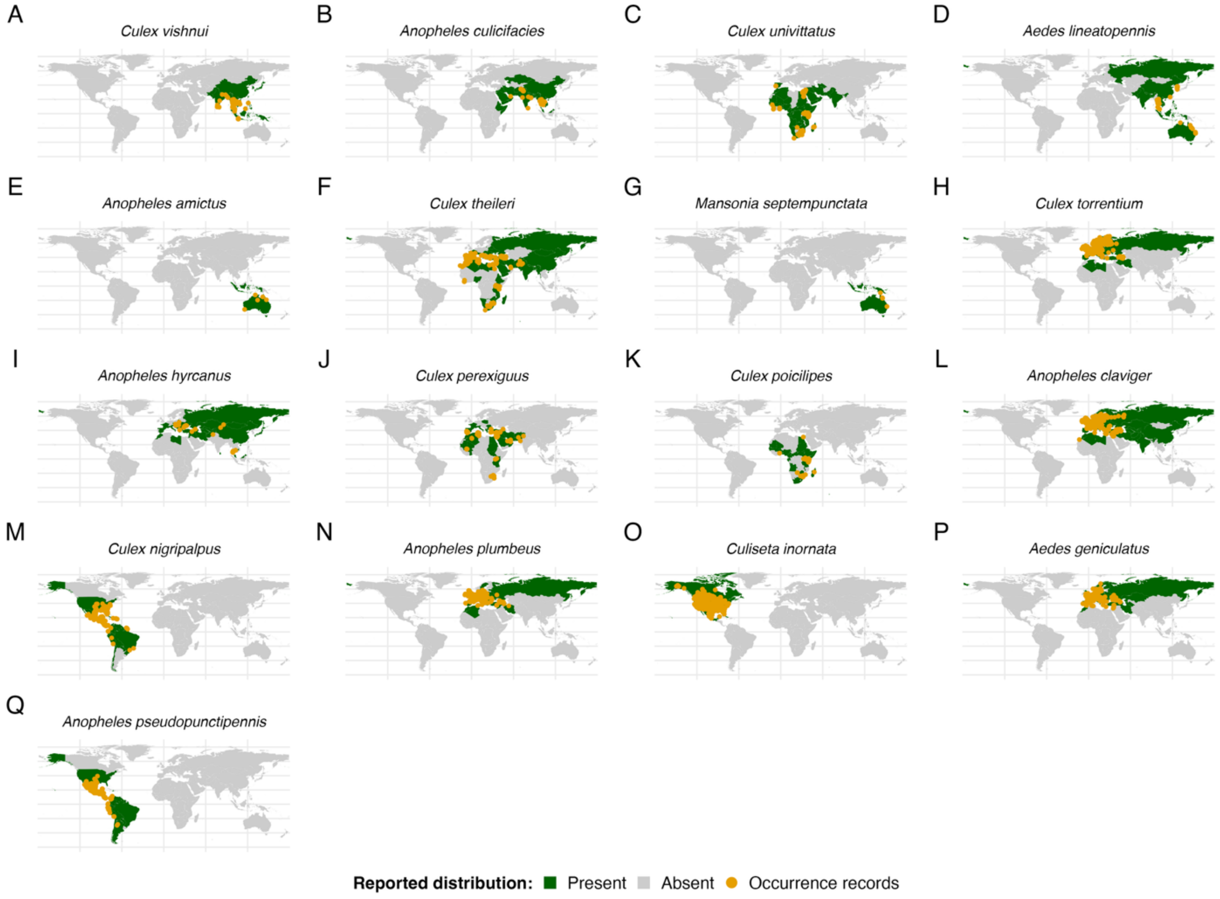

Figure B. Current reported distributions of species with high predicted invasion potential by the models including the full set of trait variables, defined as species with above-threshold ensemble predictions for all four response varibales. Green shading shows countries where each species has been reported according to Wilkerson et al. (1). Yellow points show occurrence records from gbif (2) and VectorMap (3). Base map layer adapted from GADM version 4.1 under a non-commercial academic license (https://gadm.org/license.html).

Results of models without biogeographical variables

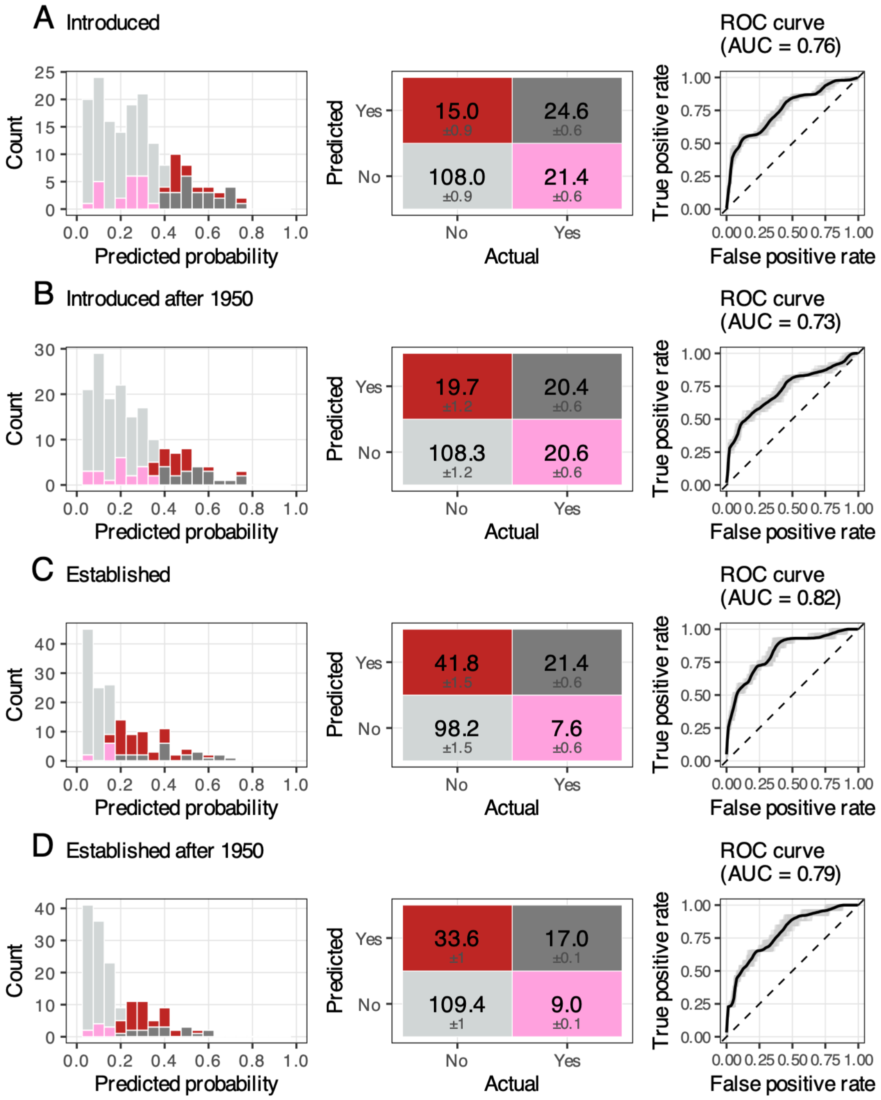

Figure C. LOOCV performance of random forest models excluding biogeographic predictors, defined here as native continent and native-range size. Panels show (a) introduction (AUC = 0.76), (b) introduction after 1950 (AUC = 0.73), (c) establishment (AUC = 0.82), and (d) establishment after 1950 (AUC = 0.79). For each model, panels show predicted probability distributions, mean ± SD confusion matrices, and ROC curves across 100 repetitions, with the average ROC curve shown in black. Red indicates false positives and pink indicates false negatives.

Table D. LOOCV random forest model performance for models excluding biogeographic predictors. Values show mean ± SD across 100 model repetitions for models predicting introduction, introduction after 1950, establishment, and establishment after 1950.

Response	AUC	OOB error	Threshold tss	Accuracy	Sensitivity	Specificity	Precision	F1
Introduced	0.76±0	0.16±0	0.39±0.02	0.79±0.02	0.54±0.03	0.88±0.04	0.63±0.04	0.58±0.01
Introduced after 1950	0.73±0	0.16±0	0.35±0.05	0.77±0.04	0.54±0.08	0.84±0.08	0.54±0.07	0.53±0.01
Established	0.82±0	0.11±0	0.17±0.05	0.71±0.06	0.83±0.10	0.69±0.09	0.37±0.05	0.50±0.03
Established after 1950	0.79±0	0.11±0	0.20±0.05	0.74±0.07	0.70±0.10	0.75±0.10	0.35±0.05	0.46±0.03

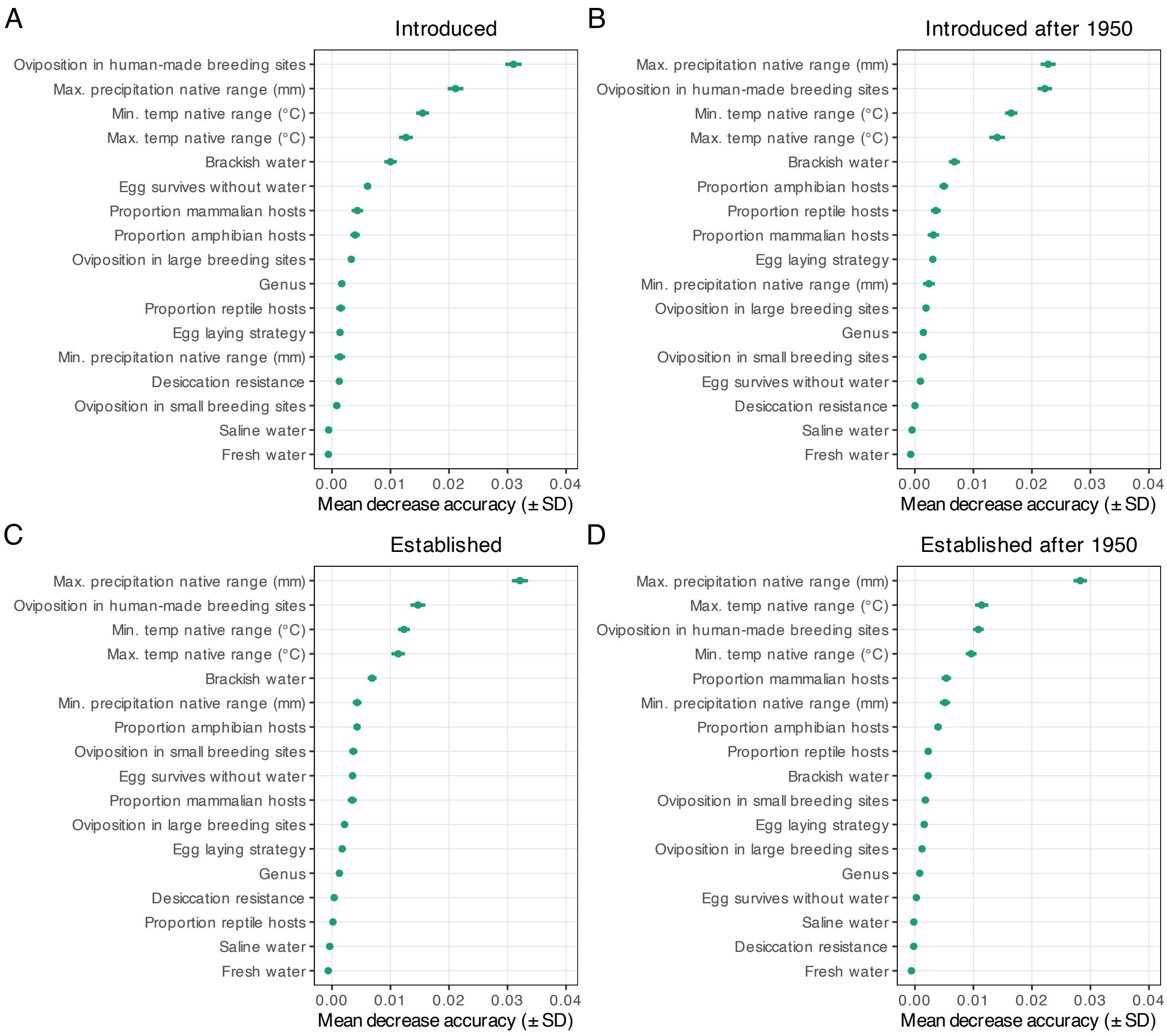

Figure D. Variable importance rankings from the random forest models without biogeographic predictors. Points show mean decrease in accuracy ± SD across 100 model repetitions. Higher values indicate greater predictor importance for (a) introduction, (b) introduction after 1950, (c) establishment, and (d) establishment after 1950.

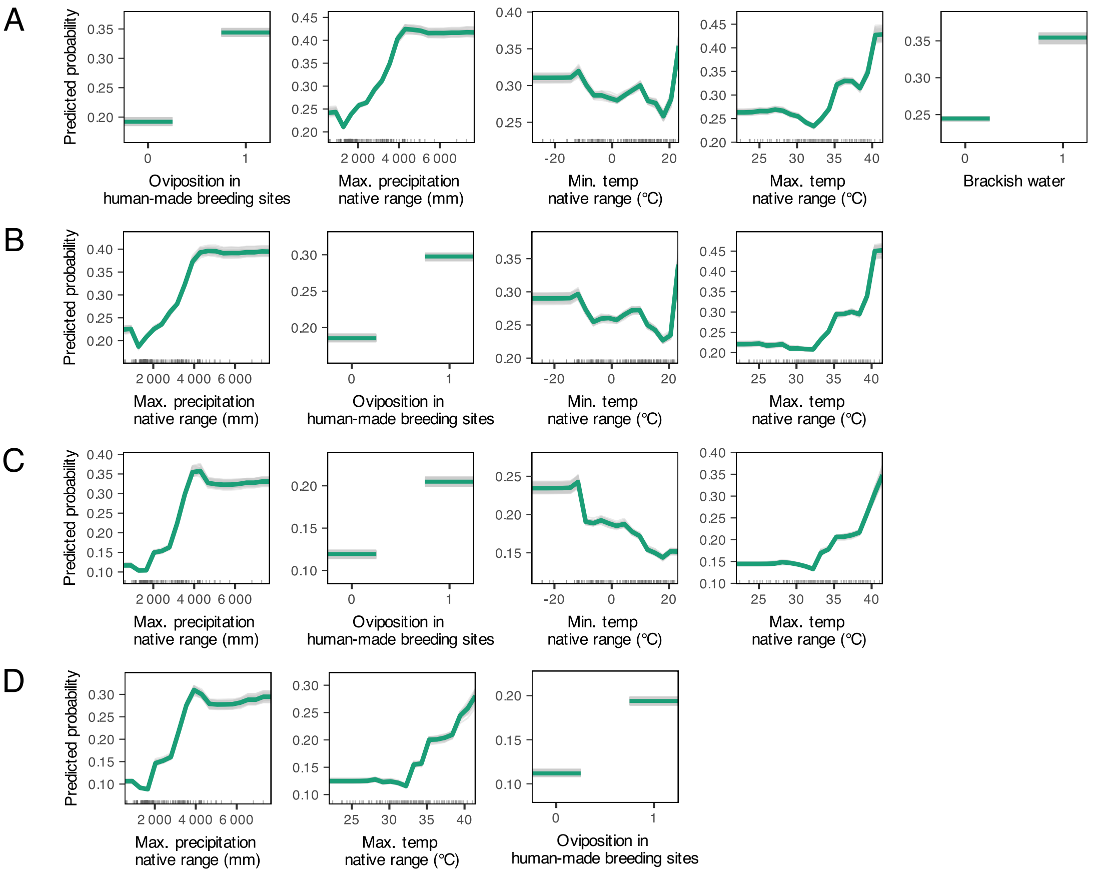

Figure E. Partial dependence plots from models without biogeographic predictors. Plots show the marginal effects of predictors with importance ≥ 0.01 on predicted probabilities. Grey lines show individual model repetitions and green lines show the average response across 100 repetitions for (a) introduction, (b) introduction after 1950, (c) establishment, and (d) establishment after 1950.

Table E. Ensemble-predicted probabilities for false-positive species in the models without biogeographic predictors. False positives are species with no recorded positive invasion status for a given response variable but with ensemble mean predicted probabilities above the corresponding optimal-TSS threshold. Values show ensemble mean predicted probabilities ± SD across 100 model repetitions. NA indicates that the species was not classified as a false positive for that response variable. Species in **bold** were identified as "most likely future invaders" only in the models without biogeographic predictors, but not in the full models in the main text. * indicate predictions exceeding the highest predicted probability among true positives for that response variable.

Species	Introduced	Introduced post-1950	Estalished	Established post-1950
Culex vishnui	0.76 ± 0.01*	0.75 ± 0.01	0.50 ± 0.01	0.44 ± 0.01

Culex nigripalpus	0.63 ± 0.01	0.61 ± 0.01	0.49 ± 0.02	0.31 ± 0.01
Anopheles culicifacies	0.61 ± 0.01	0.51 ± 0.01	0.41 ± 0.01	0.45 ± 0.02
Anopheles nuneztovari	0.57 ± 0.02	0.50 ± 0.01	0.33 ± 0.01	0.33 ± 0.01
Anopheles pseudopunctipennis	0.48 ± 0.01	0.40 ± 0.02	0.18 ± 0.01	0.24 ± 0.01
Anopheles grabhamii	0.48 ± 0.02	0.39 ± 0.02	0.17 ± 0.02	NA
Aedes africanus	0.47 ± 0.02	0.43 ± 0.02	0.45 ± 0.02	0.42 ± 0.01
Culex perexiguus	0.47 ± 0.02	0.49 ± 0.01	0.26 ± 0.01	0.23 ± 0.01
Culex theileri	0.46 ± 0.01	0.45 ± 0.01	0.31 ± 0.02	0.28 ± 0.01
Culiseta inornata	0.46 ± 0.01	0.48 ± 0.01	0.41 ± 0.02	0.39 ± 0.01
Culex torrentium	0.46 ± 0.02	0.44 ± 0.02	0.32 ± 0.01	0.32 ± 0.01
Aedes taeniorhynchus	0.45 ± 0.02	NA	0.35 ± 0.01	0.29 ± 0.01
Culex univittatus	0.44 ± 0.02	0.45 ± 0.01	0.35 ± 0.01	0.32 ± 0.01
Aedes geniculatus	0.40 ± 0.02	NA	0.25 ± 0.01	0.23 ± 0.01
Aedes argenteopunctatus	NA	0.38 ± 0.01	0.32 ± 0.01	0.27 ± 0.01
Aedes togoi	NA	0.49 ± 0.02	NA	0.40 ± 0.01
Anopheles funestus	NA	0.46 ± 0.01	0.39 ± 0.01	0.29 ± 0.01
Anopheles hyrcanus	NA	0.35 ± 0.01	0.29 ± 0.02	0.24 ± 0.01
Culex salinarius	NA	0.37 ± 0.02	NA	NA
Mansonia africana	NA	0.36 ± 0.01	NA	NA
Psorophora confinnis	NA	0.39 ± 0.01	0.40 ± 0.02	0.42 ± 0.01
Aedes luteocephalus	NA	NA	0.25 ± 0.01	0.27 ± 0.01
Aedes pembaensis	NA	NA	0.18 ± 0.01	NA
Aedes polynesiensis	NA	NA	0.30 ± 0.01	0.24 ± 0.01
Aedes scapularis	NA	NA	0.27 ± 0.01	0.27 ± 0.01
Aedes scutellaris	NA	NA	0.23 ± 0.01	0.22 ± 0.01
Aedes sexlineatus	NA	NA	0.18 ± 0.01	NA
Aedes sollicitans	NA	NA	0.17 ± 0.01	NA
Aedes taylori	NA	NA	0.19 ± 0.01	NA
Aedes triseriatus	NA	NA	0.39 ± 0.02	0.35 ± 0.01
Anopheles aquasalis	NA	NA	0.23 ± 0.01	NA
Anopheles barbirostris	NA	NA	0.31 ± 0.01	0.28 ± 0.01
Anopheles claviger	NA	NA	0.21 ± 0.01	0.20 ± 0.01
Anopheles melas	NA	NA	0.22 ± 0.01	NA
Anopheles plumbeus	NA	NA	0.27 ± 0.01	0.25 ± 0.01
Anopheles quadrimaculatus	NA	NA	0.19 ± 0.01	NA
Anopheles triannulatus	NA	NA	0.19 ± 0.01	NA
Culex annulirostris	NA	NA	0.30 ± 0.01	0.31 ± 0.01
Culex antennatus	NA	NA	0.43 ± 0.01	0.28 ± 0.01
Culex bitaeniorhynchus	NA	NA	0.62 ± 0.01	0.57 ± 0.01
Culex poicilipes	NA	NA	0.19 ± 0.01	NA
Culex restuans	NA	NA	0.22 ± 0.01	0.22 ± 0.01
Culiseta morsitans	NA	NA	0.22 ± 0.01	0.22 ± 0.01
Haemagogus mesodentatus	NA	NA	0.29 ± 0.02	NA

Limatus asulleptus	NA	NA	0.17 ± 0.01	NA
Psorophora albigenu	NA	NA	0.18 ± 0.01	NA
Anopheles arabiensis	NA	NA	NA	0.42 ± 0.01
Culex sitiens	NA	NA	NA	0.41 ± 0.01

Table F. Known invaders not captured by models without biogeographic predictors. False negatives are species with confirmed introduction or establishment records, but ensemble mean predicted probabilities below the corresponding TSS-optimal threshold. Values show ensemble mean predicted probabilities ± SD across 100 model repetitions. NA indicates that the species was not classified as a false negative for that response variable.

Species	Introduced	Introduced post-1950	Established	Established post-1950
Aedes camptorhynchus	0.12 ± 0.01	0.10 ± 0.01	NA	NA
Aedes japonicus	0.30 ± 0.01	0.14 ± 0.01	0.15 ± 0.01	0.09 ± 0.01
Aedes mcintoshi	0.09 ± 0.01	0.08 ± 0.01	NA	NA
Aedes notoscriptus	0.27 ± 0.03	0.21 ± 0.02	0.13 ± 0.01	0.11 ± 0.01
Aedes scutellaris	0.25 ± 0.01	NA	NA	NA
Aedes vexans	0.24 ± 0.01	0.22 ± 0.01	0.15 ± 0.01	0.16 ± 0.01
Aedes vigilax	0.24 ± 0.01	0.21 ± 0.01	0.04 ± 0.01	0.04 ± 0.01
Aedes vittatus	0.29 ± 0.01	0.28 ± 0.01	NA	NA
Anopheles bancroftii	0.11 ± 0.01	0.10 ± 0.01	0.05 ± 0.01	0.06 ± 0.01
Anopheles barbirostris	0.31 ± 0.01	0.28 ± 0.01	NA	NA
Anopheles coustani	0.37 ± 0.02	NA	NA	NA
Anopheles crucians	0.10 ± 0.01	0.06 ± 0.01	NA	NA
Anopheles darlingi	0.31 ± 0.02	0.20 ± 0.01	0.16 ± 0.01	0.10 ± 0.01
Anopheles gambiae s.s.	NA	0.29 ± 0.01	NA	0.15 ± 0.01
Anopheles litoralis	0.30 ± 0.02	0.26 ± 0.02	NA	NA
Anopheles maculipennis	0.18 ± 0.01	NA	NA	NA
Anopheles pharoensis	0.12 ± 0.01	0.06 ± 0.01	NA	NA
Anopheles punctipennis	0.22 ± 0.01	0.19 ± 0.02	NA	NA
Culex coronator	0.30 ± 0.01	0.33 ± 0.01	NA	NA
Culex modestus	0.27 ± 0.01	0.23 ± 0.01	0.14 ± 0.01	0.13 ± 0.01
Culex pipiens s.s.	NA	0.32 ± 0.02	NA	NA
Culex tarsalis	NA	0.33 ± 0.01	NA	NA
Culiseta annulata	0.24 ± 0.01	0.19 ± 0.01	0.14 ± 0.01	0.11 ± 0.01
Mansonia titillans	0.05 ± 0.01	0.06 ± 0.01	NA	NA

References

1. Wilkerson RC, Linton YM, Strickman D. Mosquitoes of the World. 1st edition. Baltimore, Maryland: Johns Hopkins University Press; 2021. 1332 p.

2. GBIF. Global Biodiversity Information Facility - Free and open access to biodiversity data [Internet]. 2025 [cited 2025 Apr 17]. Available from: https://www.gbif.org/

3. WRBU. (Walter Reed Biosystematics Unit) VectorMap Data Portal. VectorMap Website [Internet]. 2024 [cited 2024 Dec 17]. Available from: https://arcg.is/q5SW5
