# Supplementary material for: Trait-based prediction of disease-vector mosquito invasion potential
Source: PLoS Negl Trop Dis. 2026 Jul 23;20(7):e0014538. doi: 10.1371/journal.pntd.0014538 (PMC13395358; doi:10.1371/journal.pntd.0014538)
Supplement: Attachment — Submitted filename: Response to the reviewers.pdf [file pntd.0014538.s004.pdf]

Response to the editor

Lisbon, May 25, 2025

Clarence Mang'era
Academic Editor
PLOS Neglected Tropical Diseases

Editor Comments

1. Explicitly state which known invasive mosquito species were included in model training, clarify species inclusion criteria for the establishment models, and preferably provide a complete list of all assessed species in the Supplementary Information.
2. Expand the discussion regarding the use of broad geographic/continental predictors, particularly the role of Asia as a source region, and acknowledge that these patterns may reflect historical propagule pressure rather than future invasion dynamics.
3. More clearly identify false negatives, species with high predicted invasion potential Revise relevant tables for clarity and interpretability.
4. Clarify how replicate model outputs and uncertainty were summarised, briefly explain performance metrics (AUC, TSS), and address the editorial and readability issues raised by the reviewers (Improve definition and interpretation of terms such as “future spreaders” and similar potentially ambiguous terms).
5. The post-1950 analyses may remain but should be more clearly justified and streamlined where possible.
6. Streamline or shorten several long paragraphs in the Introduction and Discussion such as in line 388.

R#1: Dear Dr. Mang'era, we appreciate the opportunity to submit a revised version of our manuscript. We have addressed all comments and suggestions raised by the editor and the three reviewers, whose careful feedback we are very grateful for. Their comments have helped us improve the clarity, structure and interpretation of the manuscript.

The main revisions, and following your indications, include the following:

1. We now add a supplementary table (Appendix S2 Table 1) listing all species included in the models together with their binary status for introduction, introduction post-1950, establishment and establishment post-1950.
2. We also expanded the discussion on the use of broad geographic predictors (Line 436 onward), particularly native continents and clarified that they reflect historical and current propagule pressure and trade pathways rather than fixed future invasion dynamics.
3. We now also provide a clearer justification for the inclusion of the post-1950 models (L227-235), emphasizing that they likely provide a better representation of recent historical human-mediated transportation dynamics.
4. We also discuss false negatives and false positives now in more detail (L398-407). In addition, Table 2 now reports predicted probabilities of all false positive species from all four response variables, and Table 3 summaries predicted probabilities for false negative species.
5. We now also include more in-depth explanations of model evaluation metrics including AUC, TSS (L271 onward) and of how ensemble predictions and uncertainty were summarized (L284-288).
6. We revised the ambiguous term “future spreaders”, which can easily be mistaken for disease spread, to "species with high predicted invasion potential".
7. We also streamline several sections of the introduction and discussion to improve readability.

We believe these and the below described changes have improved the quality and clarity of the manuscript. All additions and modifications to the text are highlighted in blue to facilitate tracking.

We thank you for your consideration of our improved manuscript and look forward to your feedback.

Kind regards,
Rebecca Pabst on behalf of all co authors

Responses to the reviewers

Reviewer #1
Methods:
Results:
Conclusion:
General Comments:
MAJOR COMMENTS

(1) I think their traits-only model is more ecologically defensible than their traits + macroecology model. Their main discussion on their model's caveat supports this --- that biogeographic information proxies current and past propagule pressure, but it is unknown if this pattern of propagule pressure will persist in the future. If we really want the predictions to be future-thinking, then biogeographic predictors should be removed. This also solves the issues of time (the 1950 splits) --- if there are truly intrinsic traits, then this should be timeless.

As a compromise, present the predicted probabilities as a column in Table 3.
R#2: We thank the reviewer for this important point. We agree that the use of broad biogeographic predictors requires careful interpretation, as these variables may reflect the ways humans interact with and transport species (determining propagule pressure), rather than purely intrinsic species properties.

However, we believe that including these macroecological predictors remains important because the role of intrinsic species traits is itself not independent of human dynamics. In other words, even models based exclusively on intrinsic traits implicitly assume the continuation of the types and intensities of human activities occurring during the period represented in the data. For example, traits such as egg desiccation resistance are only likely to remain important predictors of invasion success as long as transport pathways continue to expose mosquitoes to dry transport conditions, such as those associated with used tires. Thus, trait-only models are also contingent on historically specific human-mediated introduction and transport processes.

Accordingly, (and also in line with the Editor's indications; see R#1) we opted to retain the models including macroecological predictors in combination with intrinsic traits in the main manuscript, as these capture both the species-specific component and the human-interaction component of invasion dynamics. These models also achieved substantially higher predictive performance (AUC) and are informative for current and near-term surveillance under present-day trade conditions. However, to address the reviewer's concern, we retained the models based exclusively on intrinsic traits, excluding biogeographic predictors, in Appendix S2, and added the predicted probabilities of all models for species with high predicted invasion potential (Table 2, L409-417) while expanding their interpretation in the Results and Discussion sections (L371-373, L463-467).

(2) The post-1950s models contribute nothing but potential source of confusion. Omit this from main text and place in Supporting information.
R#3: We thank the reviewer for raising this issue and agree that the post-1950 models required clearer justification. The rationale for developing these models follows directly from the argument outlined above (R#2): any trait-based assessment of invasion potential is inherently contingent on the characteristics of human-mediated transport and trade systems. Because these systems change through time — including transportation speed, connectivity, traded commodities, and movement pathways — the traits associated with invasion success are also expected to change over time.

Accordingly, we developed post-1950 models to better capture the introduction dynamics operating under contemporary globalization conditions and, therefore, those most likely to remain relevant in the near future. For example, traits favoring persistence during long and slow transport processes may have been more important historically than under current high-speed and highly connected transportation networks.

The post-1950 threshold was not selected ad hoc, but because it broadly corresponds to the onset of the “Great Acceleration” in global trade, transport, and human connectivity, a period strongly associated with the rapid increase in the number and diversity of biological invasions worldwide, particularly among insects (Seebens et al., 2017; Bertelsmeier et al., 2025).

We therefore argue that the post-1950 models are theoretically more appropriate for representing current and near-future invasion dynamics. In contrast, models calibrated using pre-1950 introductions likely reflect substantially different transportation systems and propagule pathways, potentially emphasizing traits that are less relevant under present-day introduction conditions.

We extended the explanation in the methods section 2.3 to (L227-235): “The year 1950 was used as an additional temporal threshold because it broadly marks the onset of the post-World War II ‘Great Acceleration’ in global trade, transportation, and human connectivity, a period associated with major increases in the number and diversity of biological invasions (44–46). Because invasion processes are strongly shaped by human-mediated transport dynamics, trait associations inferred from older introduction events may not fully represent the mechanisms driving contemporary invasions. The post-1950 models were therefore developed to better capture the transport and introduction dynamics operating under current globalization conditions and to provide invasion predictions more directly relevant to present-day and near-future introduction processes.”

(3) What can be said about species with high introduction probability but low establishment probability?
R#4: Conceptually, these are species possessing traits that increase their probability of being introduced into non-native regions, while not enhancing, or potentially even limiting, their likelihood of successful establishment following introduction. In other words, some traits may favor transport and introduction through human-mediated pathways, but not necessarily persistence or population growth under the environmental conditions encountered after introduction. These, in principle, should be of lower priority for surveillance than species having high introduction and establishment probability.

We now mention them explicitly in the Results text (L395-397) and list them in Table 2:
“On the other hand, species exceeding the introduction threshold but not the establishment threshold may possess traits favoring transport and introduction, but not long-term establishment under non-native ecological or climatic conditions.”

(4) What can be said about the false negatives? if we want to follow a precautionary approach, maybe understanding these outlier profiles may be useful, too?
R#5: We now mention the false negatives specifically in the methods, listed them specifically in Table 3 and expanded the results accordingly (L398-407):
“Some species with known invasion histories were not correctly classified by one or more models (Table 3). The false negatives had confirmed introduction or establishment records but predicted probabilities below the corresponding optimal TSS thresholds, indicating cases where invasion history was not fully explained by the available ecological, life-history, and

macroecological traits. Most false-negative species showed limited invasion success: seven out of the 15 species had been introduced but had no confirmed establishment, and six had established in only one or two regions. These cases suggest that many false negatives may reflect transient introductions or failure to establish self-sustaining populations, suggesting stochastic introduction events or influences from drivers beyond trait-based predictors, such as chance transport or climatic anomalies, or traits not captured in the dataset."

POINT-BY-POINT COMMENTS:

"... . However, in recent decades, the range of several mosquito vectors has expanded at an unprecedented pace. ..."
Comment : should you also mention whether this is naturally or/and in combination with human-mediated dispersal and spread?
R#6: We revised the sentence to specify human-mediated introduction (L54-56):
"However, in recent decades, the range of several mosquito vectors has expanded at an unprecedented pace, due to human-mediated introduction to new regions. "

"... These introductions have expanded the species' ranges, sometimes into new continents and other fairway regions and enabled local disease transmission in areas considered unsuitable or free of certain diseases, a reminder that while mosquitoes can exist without transmitting pathogens, such pathogens cannot be without mosquitoes (6,7). ..."
Comment : Complex sentence. Please break this down.
R#7: We split and simplified the sentence (L59-62):
"These introductions have expanded the species' ranges, sometimes into new continents and other distant regions. In several instances, they have also enabled local transmission of mosquito-borne pathogens in areas previously considered free of these diseases or unsuitable for sustained transmission (6,7)."

"... Despite ..."
Comment : This is a very long paragraph. Break into two please.
R#8: Thanks for the comment, we split the paragraph.

Page 5:
"... o address this question, ..."
Comment : You can break the paragraph here. Also, this new paragraph disrupts the flow of thought ---
You can perhaps put this after the objectives paragraph --- making a prediction, and this being the explanation of your predictions. OR, since it describes why specific traits were selected, maybe put this in the methods (i.e., it is a variable choice rationale).
R#9: We broke the paragraph to improve readability. We agree that the paragraph disrupted the flow of thought and have moved a shorter version of the content as suggested to the methods section (L126-135).

Page 5:
"... In order to provide warning I ..." Comment : You can also break the paragraph further here.
R#10: Thanks! Done accordingly.

Page 7:
"... Table 1. ..."
Comment : I would put this as an appendix.
R#11: As suggested, the table was moved to the Supplementary Information.

Page 13:
"... . We developed four RF models to 202 assess the invasion potential of mosquito species. The first model estimated the probability of a 203 species being introduced outside its native range, and the second the probability of the species 204 being introduced after 1950. The third and fourth models evaluated the probability of species 205 establishing themselves outside their native range (i.e., forming self-sustaining populations after 206 their introduction), considering either all records, or only those establishments occurring after 1950 207 respectively ..."

Comment : For the establishment models, did you only include species with introduction history? i.e., species with no introduction history was removed from the model?

After reading the entire paper, I do not see any value in splitting the dataset by pre-and post-1950. Remove this as this does not contribute much except for confusion.
R#12: We clarified the model inclusion criteria and included the Table Appendix S2 Table 1 that lists the four binary response variables. We retained the post-1950 models (please see our rationale in R#3 above; and also following indication from the editor, please see R#1) but revised the Methods and Discussion (See L227-235, L455-457) and added the predicted probabilities of the post-1950 models in the Results to Table 2.

Page 13:
"... Introduction and establishment dates were derived from Pabst et al. 211 (5). ..."
Comment : how many species have a history of introduction, and a history of establishment?
R#13: We added the requested numbers to the Methods (L223-226):
"The introduction model included 46 species coded as introduced, and the establishment model included 29 species coded as established. Restricting the analyses to post-1950 invasion events resulted in 41 and 26 species being classified as introduced and established, respectively."

As already mentioned, we now also add a supplementary table listing all modelled species and their four binary response variables (please see in Appendix S2 Table 1)

Page 15:
"... Figure 1. ..."
Comment : This is not really very relevant to the MS. So better put this in supporting information.
Alternatively, it would be better to have a diagram of your methods.
R#14: As suggested, we moved the original figure to the Supplementary Information (Appendix S2 Figure 1) and added a workflow diagram of the analytical approach (new Figure 1).

Page 15:
"... For each response variable, the 100 imputed datasets produced in section 2.2 were used to train 233 100 separate RF models having the same parameters, implemented in the 'ranger' package (49). ..."

Comment : What's missing later is how the predictions of each RF model were averaged, and variance statistics (SD) were used as a measure of uncertainty brought by the imputation process.
R#15: We thank the reviewer for this comment and have clarified the methodological approach. We expanded section 2.5 to describe how predictions were averaged across the 100 RF model repetitions and how SD was used to summarize uncertainty associated with imputation and model repetition (please see from L284 onward).

Page 16:
"... e area under the receiver operating characteristic curve (AUC; 52 ..."
Comment : How are the values of the AUC interpreted?
R#16: We now added an explanation of AUC in section 2.5, including that AUC measures discrimination ability and that values closer to 1 indicate stronger discrimination, whereas 0.5 indicates performance no better than random (L271-273).

Page 16:
"... True Skill 251 Statistic (TSS; 53), ..."
Comment : How are the values of TSS interpreted?
R#17: We added an explanation of TSS in sections 2.5 and 2.7, including how the TSS-optimal threshold was used to classify species and identify true positives, true negatives, false positives, and false negatives (L281-283, L303-315).

Page 16:
"... Predicted probabilities were then averaged across all 100 RF model repetitions to obtain 255 stable ensemble estimates of introduction and establishment probabilities. ..."
Comment : Also mention that you also took the variance (SD) as a measure of uncertainty brought by the imputation process.
R#18: As suggested, we now added this information to section 2.5 (L285-287).

Page 18:
"... Overall, the establishment models slightly outperformed the introduction models, reflecting 284 greater consistency in identifying traits linked to persistence after arrival. The models correctly 285 identified 35.3 of 46 species known to be introduced beyond their native ranges and 24.1 of 29 286 established species. For the post-1950 subset, 29.8 of 41 introduced and 21.6 of 26 established 287 species were accurately predicted (Figure 2). ..."
Comment : Remove.
R#19: Thanks! Done accordingly.

Page 18:
"... Table 2. LOOCV random forest model performance metrics (mean ± SD) based on 100 replicates predicting species 289 introduction and establishment probabilities. ..."
Comment : Place AUC at the very beginning. Also, there are so many metrics included here that were not mentioned in the methods. I suggest placing this table in the Supporting information, and only presenting a subset of the table containing the key evaluation metrics: AUC, TSS, OOB error.
I also think F1 is great to include, tho you then have to mention this in text.
R#20: As suggested, we revised the performance table to focus on the key metrics and placed AUC at the beginning. Additional metrics were moved to the Supplementary Information. Although informative, the F1 score was moved to the Supplementary

Information to keep the main table focused on the primary evaluation metrics and facilitate interpretation.

Page 19:
"... Figure 2. ..."
Comment: I think what is most interesting here are columns B (and maybe column A, but not column C as this is already presented in the table 1).

To further complement the results text, highlight the False positives --- these are those are the potential invaders. One way to do this is giving it a red color, and the others (TP, TN, FN) are in shades of grey.
R#21: We thank the reviewer for these suggestions. We revised the figure colors to highlight false positives in red and false negatives in pink, with true positives and true negatives shown in grey tones. We retained the ROC panels because they provide a graphical overview of model discrimination across repetitions.

Page 20:
"... Results based only on ecological and life-history traits were similar in terms of 313 identified and ranking of important variables and the predicted probabilities for the introduction 314 and establishment of species ..."
Comment : Which is only the oviposition in human-made breeding sites?
R#22: We now clarified that the reduced models excluded native continent and native-range size, not all macroecological predictors, and therefore refer to them as biogeographic variables (L247). Shared important variables included oviposition in human-made breeding sites, maximum precipitation, and temperature-related predictors, depending on the response model (Appendix S2 Figure 4+5).

Page 20:
"... preaders t ..."
Comment : ? you only modelled introduction and establishment.
R#23: We revised the terminology and now use the less ambiguous term “species with high predicted invasion potential” only as a model-based shorthand for species with high predicted probabilities of both introduction and establishment.

Page 20:
"... were not identified by the full 316 models based on all variables (Appendix S2). ..."
Comment : Can this be a problem?
R#24: We revised the sentence in the results (L371-373) to:
“Models excluding biogeographic predictors showed similar predicted probabilities and rankings of important variables. However, their performance was generally lower than that of the models with the full set of traits (AUC = 0.76-0.82; Appendix S2).“

Page 21:
"... Figure 3. ..."
Comment : I would make 2 plots: A for both all and post 1950 introductions, the two classes separated by colors. Similarly, B for both all and post 1950 establishments, the two classes separated by colors.
This would give better contrast on how these two temporally delineated models for intro and establishemnt compared.

R#25: We thank the reviewer for this helpful suggestion. However, combining variable-importance plots across models would require imposing a common predictor ordering, despite the fact that variable importance rankings differ substantially among models. Because random forest importance values are primarily interpreted relative to other predictors within the same model, such a combined representation would be more difficult to interpret model-specific importance patterns. We therefore retained separate panels to preserve the internal ranking structure and improve interpretability.

Page 23:
"... them ..."
Comment : them,
R#26: Thanks! Done

Page 23:
"... These include Culex vishnui, Anopheles culicifacies, Culex 345 univittatus, Aedes lineatopennis, Anopheles amictus, Culex theileri, Mansonia septempunctata, 346 Culex torrentium, Anopheles hyrcanus, Culex perexiguus, Culex poicilipes, Anopheles claviger, 347 Culex nigripalpus, Anopheles plumbeus, Culiseta inornata, Aedes geniculatus, and Anopheles 348 pseudopunctipennis. ..."
Comment : Then cite the table --- don't enumerate the scientific names here anymore.
R#27: We removed the long species list and referred to the table instead.

"... These species include Anopheles rufipes, Anopheles fluviatilis, 353 Coquillettidia linealis, Coquillettidia richiardii, Anopheles sergentii, Anopheles pulcherrimus, and 354 Anopheles quadrimaculatus. ..."
Comment : Then cite the table --- don't enumerate the scientific names here anymore.
R#28: As for R#27, we have removed the species names here.

Page 23:
"... Eleven species with known invasion history were not captured by our introduction model 356 (predicted probabilities < 0.3). The same applies to five species in the establishment model. Most 357 of these species exhibited transient detection or failure to establish self-sustaining populations, 358 suggesting stochastic introduction events or influences from drivers beyond trait-based predictors, 359 such as chance transport or climatic anomalies. ..."
Comment : These are also informative --- what traits do they deviate in most? for a start, are they Asian species? if not, then maybe "biogeography" (as opposed to macroecology) is not a useful trait (which I think is not). I.e., is there reason to believe that only "Asian" species are more likely to be invasive? And what's with being from asia that makes them more invasive?

Or another way of thinking this is --- sure maybe historical invaders are from Asia, but with the integration of new source regions, maybe african species will be the next emerging alien species. Unfortunately, in using Asian as a variable (and was even the strongest variable), the african potential invaders won't be detected by the model.

After reading the rest of the manuscript, I realised you acknowledge this major problem. See major comments and recommendations above.
R#29: We thank the reviewer for this comment and agree and expand the Results and Discussion section. We now discuss false negatives as informative outlier profiles (L398

onwards, Table 3, see R#5). We also expanded the discussion of native continent as a broad proxy for historical source-region dynamics and propagule pressure. We clarify that the Asia signal likely reflects past and current invasion opportunities under existing trade patterns, but that future source regions may change as trade routes and commodity flows shift. We emphasize that our model results are most relevant for current and near future surveillance (L436-457) (see also R#2 above).

Page 24:
"... Table 3. ..."
Comment : "TRUE LABEL" and "PREDICTED LABEL" column doesnt make sense --- they are all nos and yes. I suggest showing predicted probabilities for introduction and establishment for full models and traits only models.
R#30: We removed these columns and revised the tables to show ensemble predicted probabilities ± SD not only for the introduction model predictions but for all four response variables. Results from models excluding biogeographic predictors are provided in the Supplementary Information Appendix S2.

Page 25:
"... 364 Occurrence of possible spreaders ..."
Comment : Can you also plot the occurrence data? because country boundaries are not really biogeographical.
R#31: As suggested, we now add occurrence points to the distribution maps, however we moved the whole section to the Supplementary Material (Appendix S2 Figure 2).

Page 26:
"... his pattern 392 reflects Asia's role as principal source region for invasive species overall (60), ..."
Comment : But is there reason to believe that they will remain a future source?
R#32: We expanded the Discussion to address this point. We now clarify that Asia's importance should be interpreted as reflecting observed source-region dynamics, trade connectivity, and propagule pressure, not as a fixed prediction that future invasions will necessarily originate from Asia (also mentioned in R#29). We also acknowledge that changing trade routes, commodities, and transport hubs may alter future source regions.

Page 27:
"... Europe. ..."
Comment : Paragraph break
R#33: Thanks! Done

Page 27:
"... integrative factor ..."
Comment : more like an integrative proxy
R#34: Thanks! Done

Page 27:
"... Nevertheless, our results suggest that the intrinsic traits of a species remain 418 relatively stable predictors, independent of macroecological factors (Appendix S2). ..."

Comment : They are stable, and thus they may be less biased to biogeographical source information.
R#35: We understand the reviewer's point and agree that models based exclusively on intrinsic traits may be less influenced by biogeographic source information. However, as discussed above (R#2), we consider this distinction less straightforward because the predictive relevance of intrinsic traits is itself contingent on historically specific human-mediated transport dynamics.

It is also important to note that models using only intrinsic traits consistently showed lower predictive performance than models combining intrinsic and macroecological predictors. We therefore consider the inclusion of macroecological predictors justified, particularly because these variables likely capture important components of ongoing human–species interaction processes relevant to present-day introductions. In this context, combining intrinsic traits with macroecological, specifically biogeographic predictors is expected to provide more realistic and informative predictions of species likely to become invasive in the near future.

Page 28:
"... 5,71) ..."
Comment : also 22
R#36: Thanks! Added accordingly

Page 28:
"... thermally extreme ..."
Comment : this can be either hot or cold.... but you only mean hot
R#37: We revised the wording to "species from warmer regions" to be more specific (L490).

Page 29:
"... n. ..."
Comment : 22
R#38: Added as suggested

Page 30:
"... 24 species with no recorded 471 invasion history but trait profiles closely matching those of known invaders. ..."
Comment : What do we do with the other 7 low-establishment probability species?
R#39: These species are likely of lower priority for surveillance and prevention efforts than those ranking high for both introduction and establishment. As noted above (R#4), we interpret them as species possessing traits that may favor introduction into non-native regions, but not traits that promote successful establishment following introduction. Accordingly, although such species may occasionally be transported beyond their native ranges, they are expected to have comparatively lower probabilities of forming persistent non-native populations.

Page 31:
"... Nonetheless, the imputation process may 490 obscure subtle ecological differences that influence invasion potential ..."
Comment : but the SD of your predictions are small. So it seems the model is not sensitive to the imputation process.
R#40: We agree and revise the sentence to (L538-540):

“The standard deviations of our predictions were low, indicating that model outputs were not highly sensitive to the imputation process. Nonetheless, imputation may obscure subtle ecological differences.“

Reviewer #2

Methods: Materials, methods, and data are complete and adequate. The statistical analysis is correct. I'm not entirely sure whether multicollinear features should be removed in environmental research. But that's just a doubt I have.

Results: The results are valid and fully align with the objectives. The figures effectively illustrate the results and are sufficient in number.

Conclusion: The conclusions is relevant and supported by the data presented.

General Comments: Both the research idea and the methods used to implement it are interesting. The authors rightly note that the invasion potential of mosquitoes can only be partially predicted based on intrinsic biological and ecological traits alone. However, this direction holds great potential for subsequent research and methodological refinement. It is very important that the article has practical application – it allows the findings to be used to support early warning systems, the targeted allocation of surveillance resources, and ultimately, the reduction of the risk of emerging vector-borne diseases.

R#41: We sincerely thank the reviewer for their positive assessment of the manuscript and for recognizing the practical relevance of the study.

Regarding the comment on multicollinearity, we agree that the decision to remove correlated predictors in environmental research can depend on the modelling goal and context. In our case, we applied correlation filtering and VIF screening to reduce redundancy among predictors and improve interpretability of variable importance and partial dependence results. This is a standard practice in ecological prediction when the aim is not only prediction, but also explanation, such as identifying which traits are most consistently associated with introduction and establishment probabilities. We have retained this approach and clarified the rationale in the Methods (L238-245).

Reviewer #3

Methods: Analysis is complex and explained thoroughly.

In methods authors should specifically define what is considered macroecological data and how distinct from ecological data in general.

The methods should specifically mention that mosquitoes already known to be invasive were included in developing the model. Also, I did not find the full list of mosquitoes you tested in the model, this would be useful for inclusion somewhere.

R#42: We thank the reviewer for taking the time and reviewing our article and for the constructive suggestions provided.

As suggested, we have now added descriptions of ecological and life-history as well as macroecological traits in the methods section (L138-145):

“Ecological and life-history traits describe species-specific intrinsic attributes expected to influence transport, survival during transit, and establishment, including breeding habitat, use of human-made breeding sites, egg desiccation resistance, oviposition strategy and host-use patterns.

Macroecological traits represent the native-range context of each species, including native-range size, climatic limits, and broad biogeographic origin. These predictors were included because they are expected to capture both environmental tolerances and exposure to external factors like human-mediated transport pathways and opportunities for introduction

and thus serve as proxies of invasion history as well as current and past propagule pressure."

As suggested, we now further added a table (Appendix S2 Table 1) with a full list of species included in the model and their four binary response variables for the introduction, the introduction post-1950, establishment and establishment post-1950 models.

Results: Throughout the results, please make sure to specify in what way the variable impacted the invasive potential. For Example: in what way does max and min temp impact the invasive potential, is lower max temp better? Is higher min temp better? Etc. by carefully including that detail throughout the results it will give the reader better insight into how the variables actually impact invasive potential, especially important to readers without modeling background but interested in this topic.
Figures in the review PDF were difficult to read and small. When getting this ready for publication, large font size and higher quality images are needed.
R#43: We thank the reviewer for this helpful comment. We revised the Results section to place greater emphasis on the direction of the relationships between predictors and invasion probabilities, rather than repeating only the ranking of important variables already shown in the figures (L351-370). This should provide clearer biological interpretation and improve accessibility for readers less familiar with modeling approaches.

We also acknowledge that our figure with partial dependence plot (Fig. 4) is now provided with higher-resolution and improved contrast to enhance readability in the final publication format.

Conclusion: Conclusions are supported by the data.
Limitations are touched on, but should discuss that when predicting region of origin they generalized impact of continent on invasive potential. Should be some discussion that this is very broad characterization and subregions within each continent are very different and may differently impact invasive potential.
R#44: Thanks for this suggestion. Accordingly, we added the following sentence to the limitations paragraph to acknowledge this (L543-545):
"Third, the delineation of native origin follows a broad continental-level classification that ignores differences between subregions within individual continents, which may differentially influence invasion potential."

Editorial and Data Presentation Modification:

4. For title, suggest explicitly mentioning what is meant by trait: perhaps Bionomic Trait Based Assessment…
R#45: We thank the reviewer for this suggestion. However, we opted not to use the term "bionomic" in the title because it may be less immediately accessible to readers outside the mosquito/vector biology field. Instead, we revised the title to:

Trait-based prediction of disease-vector mosquito invasion potential

which we believe more clearly conveys the scope and approach of the study to a broader readership.

21-23. Sentence is contradictory, clarify what many expanded native ranges v.s established outside of native range, as expanding the native range would suggest they are outside their native range.
R#46: We agree that the previous version was misleading. Accordingly we changed the term spread to introduction, and expansion to invasion, as we are only assessing human introduced non-native mosquito species, no natural expansion. It now reads(L13-16): "Mosquito-borne diseases pose a growing global health threat, largely driven by the human-mediated introduction of vector species beyond their native regions. Although only a few mosquito species have historically established populations outside their native ranges, the number of such invasions has increased rapidly in recent decades."

Line 31. Is all of Asia considered? Or specific region of Asia
R#47: Our geographic classification was based on broad continental-level delineations, with all of Asia treated as a single category. We agree that finer regional subdivisions could capture additional biogeographic variation. However, this would also substantially increase the number of native-origin predictor categories included in the models, increasing model complexity and potentially reducing interpretability and robustness given the available sample size.

Line 34. Future spreaders, do you mean spreaders of disease or spreading to new areas. Suggest clarifying if meaning is to spreading to new areas, perhaps they have the highest likelihood for invasion.
R#48: We thank the reviewer for pointing this out and clarify now that we are not assessing disease spread, therefore we changed the term that we used for the species with high predicted invasion potential across models to "species with high predicted invasion potential" (L303-312).

Line 66. I think "fairway" is meant to be far away?
R#49: Indeed, however the sentence was deleted during the revision.

Line 95. Propagules is typically used in relation to plants and makes this confusing. "Viable eggs" is appropriate
R#50: We thank the reviewer for this suggestion. Although the term "propagules" is commonly associated with plants, it is also widely used in invasion biology more broadly, including for animal invasions, where it refers to any viable life stage capable of founding a new population. In mosquitoes, this may include not only viable eggs, but also larvae or transported gravid females. To improve clarity we now make this mention upon first use of the term propagule (L133-135): "and the prevalence of viable propagules (i.e., mosquito life stages capable of founding new populations, such as eggs, larvae, or gravid females) upon arrival (23)."

Line 116. Clarify are the 184 mosquitoes chosen known competent vectors or have they been detected as carrying the pathogen. Also are these species for consideration not yet found beyond their native range? I think it's important to clarify that in intro,
R#51: We thank the reviewer for this comment and clarify this now in the introduction (L56-59)
"Of the approximately 184 mosquito species, from which human pathogens have been isolated from wild-caught females (4), by now 46 have already been introduced into regions outside their native ranges, with 29 confirmed as having established populations (5) "

And describe it in more detail in the methods (L121-123):
"Species included in the analysis followed Pabst et al. (5) and were limited to 184 vector mosquitoes from which human pathogens were isolated from field-caught females, verified by at least two literature sources (4) or by one source plus a disease association recorded in Wilkerson et al. (26). The list of assessed species is provided in Appendix S1, the introduction and establishment status of species included in the modelling is provided in Appendix S2 Table 1."

Line 118. Please define macroecological data and how distinct from ecological data in general.
R#51: Please see Response R#42.
Line 152. How was native range determined and what criteria was used to omit countries from what is considered the native range.
R#52: We thank the reviewer for this comment and agree that the description given in the previous version is unclear. No country where a species is considered native was omitted. Accordingly we have expanded the section 2.1.1 Country wide distribution data and native range delineation to improve clarity on the procedure.

It now reads (L161-166): "Country-level distributions were obtained from Wilkerson et al. (20) and the Walter Reed Biosystematics Unit (27). For each species, all countries identified as non-native in the comprehensive compilation of Pabst et al. (5) were removed from these distributions. The remaining countries were treated as the species' native range and were checked for spatial consistency with additional published distribution information (e.g., 28,29)."

Methods. Were mosquitoes already known to be invasive included in the model, ie Ae. aegypti? If not how does the model predict invasive potential of known established non-native vectors. I see from the results they were included. The methods should specifically. Also, I did not find the full list of mosquitoes you tested in the model, this would be useful for inclusion somewhere.
R#53: Yes, all mosquito species previously known to have been introduced and/or established in non-native regions, including well-known invasive vectors such as Aedes aegypti, were included in model calibration. The models were then used to predict introduction and establishment probabilities for species not yet recorded as introduced or established outside their native ranges. We now clarify this more explicitly in the Methods section (L216-226; see also Response R#42).

We also now include a complete list of all species considered in the analyses, together with their predicted probabilities, in the Supplementary Material (Appendix S2 Table 1).

Line 310. Maximum precipitation of the host range? And how, more rain more invasive, or lower max precipitation more invasive potential?

Actually throughout the results, please make sure to specify in what way the variable impacted the invasive potential, like max and min temp. In what way does max and min temp impact the invasive potential, is lower max temp better? Is higher min temp better? Etc. by carefully including that detail throughout the results it will give the reader better insight into how the variables actually impact invasive potential, especially important to readers without modeling background but interested in this topic.

R#54: We thank the reviewer for raising this issue and have rewritten the results accordingly (please see from L354 onward).

Line 340. I think a list of the known invaders would be helpful for context, as appendix/supplement or as a table.
R#55: Please see Response R#42. We added a list to the Supplementary Material Appendix S2 Table 1.

Line 355. What are the 11 that failed your model? I think that is important
R#56: We thank the reviewer for this comment and agree that this came short in the previous version. We now describe them specifically (from L398-407) added Table 3 to the main text with the predicted probabilities for false negatives.

Line 384. I did not see it explicitly stated which species had higher introduction probabilities than those already introduced, it would be interesting to see which potential invaders were more likely to invade than actual invasive mosquitoes.
R#57: Accordingly, we now added an * in Table 2 to identify the species exceeding the highest probability of known invaders. The whole list of predicted ensemble probabilities ± SD (including those already introduced and established) was added in Appendix S2 Table 1 (see R#53)

Line 464, is precipitation extremes mean low or high or both? Still like a clear interaction from the model, I would think those species that experience the greatest fluctuation in precipitation (highest max and lowest min rain fall) would be a predictor of invasiveness, does the model show that? Or is it just highest rain max result in highest invasiveness?
R#58: We thank the reviewer for pointing out this ambiguity. The models only pointed out maximum precipitation in the native range as a strong predictor. We have revised the wording from “precipitation extremes” to “high maximum precipitation" where appropriate (L356 onwards).

General Comments:
I think this is a very interesting study, a large analysis with prediction of invasive potential of mosquitoes capable of transmitting human disease. Overall, I think this needs minor revision. Some areas should be clarified for what the results mean outside of the model, see comment for line 310 as example. A full list of species being assessed is needed somewhere in the paper, probably best for an appendix. I also would like the authors to explicitly list which known invasive mosquitoes didn't show as having high invasive potential from the model and describing why the model contradicts with these truly invasive mosquitoes.
R#59: We thank the reviewer for all their comments and have tried to incorporate them fully. We believe they have contributed significantly to improve the understanding of the manuscript.
