# Supplementary material for: Trait-based prediction of disease-vector mosquito invasion potential
Source: PLoS Negl Trop Dis. 2026 Jul 23;20(7):e0014538. doi: 10.1371/journal.pntd.0014538 (PMC13395358; doi:10.1371/journal.pntd.0014538)
Supplement: Peer review history

# Peer review: Decision Letter 0

**DOI:** 10.1371/journal.pntd.0014538.r001

**Authors:** Paul O. Mireji, Clarence Mang’era

12 May 2026

PNTD-D-25-02192

Trait based assessment of the invasion potential of disease vector mosquitoes

PLOS Neglected Tropical Diseases

Dear Dr. Pabst,

-->Thank you for submitting your manuscript to PLOS Neglected Tropical Diseases. After careful consideration, we feel that it has merit but does not fully meet PLOS Neglected Tropical Diseases's publication criteria as it currently stands. Therefore, we invite you to submit a revised version of the manuscript that addresses the points raised during the review process.

Please submit your revised manuscript by Jul 11 2026 11:59PM. If you will need more time than this to complete your revisions, please reply to this message or contact the journal office at plosntds@plos.org. When you're ready to submit your revision, log on to https://www.editorialmanager.com/pntd/ and select the 'Submissions Needing Revision' folder to locate your manuscript file.

Please include the following items when submitting your revised manuscript:

* A letter that responds to each point raised by the editor and reviewer(s). You should upload this letter as a separate file labeled 'Response to Reviewers'. This file does not need to include responses to any formatting updates and technical items listed in the 'Journal Requirements' section below.

* A marked-up copy of your manuscript that highlights changes made to the original version. You should upload this as a separate file labeled 'Revised Manuscript with Track Changes'.

* An unmarked version of your revised paper without tracked changes. You should upload this as a separate file labeled 'Manuscript'.

If you would like to make changes to your financial disclosure, competing interests statement, or data availability statement, please make these updates within the submission form at the time of resubmission. Guidelines for resubmitting your figure files are available below the reviewer comments at the end of this letter.

As the corresponding author, your ORCID iD is verified in the submission system and will appear in the published article. PLOS supports the use of ORCID, and we encourage all coauthors to register for an ORCID iD and use it as well. Please encourage your coauthors to verify their ORCID iD within the submission system before final acceptance, as unverified ORCID iDs will not appear in the published article. Only the individual author can complete the verification step; PLOS staff cannot verify ORCID iDs on behalf of authors.

We look forward to receiving your revised manuscript.

Kind regards,

Clarence Mang'era, PhD

Academic Editor

PLOS Neglected Tropical Diseases

Paul Mireji

Section Editor

PLOS Neglected Tropical Diseases

Shaden Kamhawi

co-Editor-in-Chief

PLOS Neglected Tropical Diseases

orcid.org/0000-0003-4304-636XX

Paul Brindley

co-Editor-in-Chief

PLOS Neglected Tropical Diseases

orcid.org/0000-0003-1765-0002

**Additional Editor Comments:**

The following changes should be addressed in a revised manuscript because they are important for clarity, reproducibility, and interpretation:

1. Explicitly state which known invasive mosquito species were included in model training, clarify species inclusion criteria for the establishment models, and preferably provide a complete list of all assessed species in the Supplementary Information.

2. Expand the discussion regarding the use of broad geographic/continental predictors, particularly the role of Asia as a source region, and acknowledge that these patterns may reflect historical propagule pressure rather than future invasion dynamics.

3. More clearly identify false negatives, species with high predicted invasion potential, and species exceeding known invaders in predicted probabilities. Revise relevant tables for clarity and interpretability.

4. Clarify how replicate model outputs and uncertainty were summarised, briefly explain performance metrics (AUC, TSS), and address the editorial and readability issues raised by the reviewers (Improve definition and interpretation of terms such as “future spreaders” and similar potentially ambiguous terms).

5. The post-1950 analyses may remain but should be more clearly justified and streamlined where possible.

6. Streamline or shorten several long paragraphs in the Introduction and Discussion such as in line 388.

**Journal Requirements:**

1) Please ensure that the CRediT author contributions listed for every co-author are completed accurately and in full.

At this stage, the following Authors/Authors require contributions: Rebecca Pabst, Carla A Sousa, and César Capinha. Please ensure that the full contributions of each author are acknowledged in the "Add/Edit/Remove Authors" section of our submission form.

The list of CRediT author contributions may be found here: https://journals.plos.org/plosntds/s/authorship#loc-author-contributions

2) We ask that a manuscript source file is provided at Revision. Please upload your manuscript file as a .doc, .docx, .rtf or .tex. If you are providing a .tex file, please upload it under the item type u2018LaTeX Source Fileu2019 and leave your .pdf version as the item type u2018Manuscriptu2019.

3) Some material included in your submission may be copyrighted. According to PLOS's copyright policy, authors who use figures or other material (e.g., graphics, clipart, maps) from another author or copyright holder must demonstrate or obtain permission to publish this material under the Creative Commons Attribution 4.0 International (CC BY 4.0) License used by PLOS journals. Please closely review the details of PLOS's copyright requirements here: <a data-cke-saved-href="https://journals.plos.org/plosntds/s/licenses-and-copyright" href="https://journals.plos.org/plosntds/s/licenses-and-copyright">PLOS Licenses and Copyright</a>. If you need to request permissions from a copyright holder, you may use the&nbsp;<a data-cke-saved-href="https://storage.googleapis.com/plos-published-prod/c4ff/Content%20Copyright%20Permission%20Form.pdf?X-Goog-Algorithm=GOOG4-RSA-SHA256&X-Goog-Credential=wombat-sa%40plos-prod.iam.gserviceaccount.com%2F20220817%2Fauto%2Fstorage%2Fgoog4_request&X-Goog-Date=20220817T210608Z&X-Goog-Expires=86400&X-Goog-SignedHeaders=host&X-Goog-Signature=cd9dc868773378f7033b9f8cfb91430167d6275df90bce01342a454f36512530b64f24736fbf68c4fd96da74d663b6ee95b3420bded8fb0e833af2390b896b8706643b476d7f532408921b60576d65b60617070900fd2bcd28eec4cd32fefcf007d3655df17fcc91023329a28479ce0d1c5758174eedcc9e3c1788c5f7dd8523f3ddd871e02c998a20f7dc78ec00f313d4b5a78ba2037a6c7ef0a05dc29817da9368065460c8d8446659f35f0458fefabd3101eb7f926e9cb7a0b53e7bbe47aaed3e0ba52f9af9c2b8f6d7d3a8871b205fc4cbe97384ed37c7604fd7eecfedccf49cba12820b2e3b649541cefde3103cd7d72731b3e8b0248439b4cba691ca8a" href="https://storage.googleapis.com/plos-published-prod/c4ff/Content%20Copyright%20Permission%20Form.pdf?X-Goog-Algorithm=GOOG4-RSA-SHA256&X-Goog-Credential=wombat-sa%40plos-prod.iam.gserviceaccount.com%2F20220817%2Fauto%2Fstorage%2Fgoog4_request&X-Goog-Date=20220817T210608Z&X-Goog-Expires=86400&X-Goog-SignedHeaders=host&X-Goog-Signature=cd9dc868773378f7033b9f8cfb91430167d6275df90bce01342a454f36512530b64f24736fbf68c4fd96da74d663b6ee95b3420bded8fb0e833af2390b896b8706643b476d7f532408921b60576d65b60617070900fd2bcd28eec4cd32fefcf007d3655df17fcc91023329a28479ce0d1c5758174eedcc9e3c1788c5f7dd8523f3ddd871e02c998a20f7dc78ec00f313d4b5a78ba2037a6c7ef0a05dc29817da9368065460c8d8446659f35f0458fefabd3101eb7f926e9cb7a0b53e7bbe47aaed3e0ba52f9af9c2b8f6d7d3a8871b205fc4cbe97384ed37c7604fd7eecfedccf49cba12820b2e3b649541cefde3103cd7d72731b3e8b0248439b4cba691ca8a">PLOS Content Copyright Permission&nbsp;Form</a>

Please respond directly to this email and provide any known details concerning your material's license terms and permissions required for reuse, even if you have not yet obtained copyright permissions or are unsure of your material's copyright compatibility. Once you have responded and addressed all other outstanding technical requirements, you may resubmit your manuscript within Editorial Manager.

Potential Copyright Issues:

- Figure 5: Please (a) provide a direct link to the base layer of the map (i.e., the country or region border shape) and ensure this is also included in the figure legend; and (b) provide a link to the terms of use / license information for the base layer image or shapefile. We cannot publish proprietary or copyrighted maps (e.g. Google Maps, Mapquest) and the terms of use for your map base layer must be compatible with our CC BY 4.0 license.

Note: if you created the map in a software program like R or ArcGIS, please locate and indicate the source of the basemap shapefile onto which data has been plotted.

If your map was obtained from a copyrighted source please amend the figure so that the base map used is from an openly available source. Alternatively, please provide explicit written permission from the copyright holder granting you the right to publish the material under our CC BY 4.0 license.

If you are unsure whether you can use a map or not, please do reach out and we will be able to help you. The following websites are good examples of where you can source open access or public domain maps:

* U.S. Geological Survey (USGS) - All maps are in the public domain. (http://www.usgs.gov)

* PlaniGlobe - All maps are published under a Creative Commons license so please cite u201cPlaniGlobe, http://www.planiglobe.com, CC BY 2.0u201d in the image credit after the caption. (http://www.planiglobe.com/?lang=enl)

* Natural Earth - All maps are public domain. (http://www.naturalearthdata.com/about/terms-of-use/).

4) Please amend your detailed Financial Disclosure statement. This is published with the article. It must therefore be completed in full sentences and contain the exact wording you wish to be published.

- State the initials, alongside each funding source, of each author to receive each grant. For example: "This work was supported by the National Institutes of Health (####### to AM; ###### to CJ) and the National Science Foundation (###### to AM)."

- State what role the funders took in the study. If the funders had no role in your study, please state: "The funders had no role in study design, data collection and analysis, decision to publish, or preparation of the manuscript.".

If you did not receive any funding for this study, please simply state: u201cThe authors received no specific funding for this work.u201d

**Reviewers' comments:**

Reviewer's Responses to Questions

**Key Review Criteria Required for Acceptance?**

As you describe the new analyses required for acceptance, please consider the following:

**Methods:**

-Are the objectives of the study clearly articulated with a clear testable hypothesis stated?

-Is the study design appropriate to address the stated objectives?

-Is the population clearly described and appropriate for the hypothesis being tested?

-Is the sample size sufficient to ensure adequate power to address the hypothesis being tested?

-Were correct statistical analysis used to support conclusions?

-Are there concerns about ethical or regulatory requirements being met?

Reviewer #1: (No Response)

Reviewer #2: Materials, methods, and data are complete and adequate. The statistical analysis is correct. I'm not entirely sure whether multicollinear features should be removed in environmental research. But that's just a doubt I have.

Reviewer #3: Analysis is complex and explained thoroughly.

In methods authors should specifically define what is considered macroecological data and how distinct from ecological data in general.

The methods should specifically mention that mosquitoes already known to be invasive were included in developing the model. Also, I did not find the full list of mosquitoes you tested in the model, this would be useful for inclusion somewhere.

**********

**Results**

-Does the analysis presented match the analysis plan?

-Are the results clearly and completely presented?

-Are the figures (Tables, Images) of sufficient quality for clarity?

Reviewer #1: (No Response)

Reviewer #2: The results are valid and fully align with the objectives. The figures effectively illustrate the results and are sufficient in number.

Reviewer #3: Throughout the results, please make sure to specify in what way the variable impacted the invasive potential. For Example: in what way does max and min temp impact the invasive potential, is lower max temp better? Is higher min temp better? Etc. by carefully including that detail throughout the results it will give the reader better insight into how the variables actually impact invasive potential, especially important to readers without modeling background but interested in this topic.

Figures in the review PDF were difficult to read and small. When getting this ready for publication, large font size and higher quality images are needed.

**********

**Conclusions**

-Are the conclusions supported by the data presented?

-Are the limitations of analysis clearly described?

-Do the authors discuss how these data can be helpful to advance our understanding of the topic under study?

-Is public health relevance addressed?

Reviewer #1: (No Response)

Reviewer #2: The conclusions is relevant and supported by the data presented.

Reviewer #3: Conclusions are supported by the data.

Limitations are touched on, but should discuss that when predicting region of origin they generalized impact of continent on invasive potential. Should be some discussion that this is very broad characterization and subregions within each continent are very different and may differently impact invasive potential.

**********

**Editorial and Data Presentation Modifications?**

Use this section for editorial suggestions as well as relatively minor modifications of existing data that would enhance clarity. If the only modifications needed are minor and/or editorial, you may wish to recommend “Minor Revision” or “Accept”.

Reviewer #1: (No Response)

Reviewer #2: (No Response)

Reviewer #3: Editorial suggestions:

4. For title, suggest explicitly mentioning what is meant by trait: perhaps Bionomic Trait Based Assessment…

21-23. Sentence is contradictory, clarify what many expanded native ranges v.s established outside of native range, as expanding the native range would suggest they are outside their native range.

Line 31. Is all of Asia considered? Or specific region of Asia

Line 34. Future spreaders, do you mean spreaders of disease or spreading to new areas. Suggest clarifying if meaning is to spreading to new areas, perhaps they have the highest likelihood for invasion.

Line 66. I think “fairway” is meant to be far away?

Line 95. Propagules is typically used in relation to plants and makes this confusing. "Viable eggs" is appropriate

Line 116. Clarify are the 184 mosquitoes chosen known competent vectors or have they been detected as carrying the pathogen. Also are these species for consideration not yet found beyond their native range? I think it’s important to clarify that in intro,

Line 118. Please define macroecological data and how distinct from ecological data in general.

Line 152. How was native range determined and what criteria was used to omit countries from what is considered the native range.

Methods. Were mosquitoes already known to be invasive included in the model, ie Ae. aegypti? If not how does the model predict invasive potential of known established non-native vectors. I see from the results they were included. The methods should specifically. Also, I did not find the full list of mosquitoes you tested in the model, this would be useful for inclusion somewhere.

Line 310. Maximum precipitation of the host range? And how, more rain more invasive, or lower max precipitation more invasive potential?

Actually throughout the results, please make sure to specify in what way the variable impacted the invasive potential, like max and min temp. In what way does max and min temp impact the invasive potential, is lower max temp better? Is higher min temp better? Etc. by carefully including that detail throughout the results it will give the reader better insight into how the variables actually impact invasive potential, especially important to readers without modeling background but interested in this topic.

Line 340. I think a list of the known invaders would be helpful for context, as appendix/supplement or as a table.

Line 355. What are the 11 that failed your model? I think that is important

Line 384. I did not see it explicitly stated which species had higher introduction probabilities than those already introduced, it would be interesting to see which potential invaders were more likely to invade than actual invasive mosquitoes.

Line 464, is precipitation extremes mean low or high or both? Still like a clear interaction from the model, I would think those species that experience the greatest fluctuation in precipitation (highest max and lowest min rain fall) would be a predictor of invasiveness, does the model show that? Or is it just highest rain max result in highest invasiveness?

**********

**Summary and General Comments**

Use this section to provide overall comments, discuss strengths/weaknesses of the study, novelty, significance, general execution and scholarship. You may also include additional comments for the author, including concerns about dual publication, research ethics, or publication ethics. If requesting major revision, please articulate the new experiments that are needed.

Reviewer #1: ## PNTD-D-25-02192 (2).pdf

Thank you for the opportunity to read and review the Manuscript PNTD-D-25-02192 ("MS"). The MS predicted the introduction and establishment potential of mosquitos who are vectors for human diseases and who have no history of invasion (yet). I am genuinely impressed by the amount of work that has been poured into this study, and I think it will contribute significantly to theory and application.

I outline below my major comments, followed by my point-by-point comments.

====================

Disclaimers about me that may have affected my commentary:

(1) I am not a native English speaker, so I did not comment on their grammar. However, I did comment on readability.

(2) I am a biodiversity and conservation informaticist, and my work has focused on developing tools for biodiversity assessments and conservation decision support. I have also worked on biological invasions, but not vectors of pathogens.

(3) I have worked with functional traits.

=======================

MAJOR COMMENTS

(1) I think their traits-only model is more ecologically defensible than their traits + macroecology model. Their main discussion on their model's caveat supports this --- that biogeographic information proxies current and past propagule pressure, but it is unknown if this pattern of propagule pressure will persist in the future. If we really want the predictions to be future-thinking, then biogeographic predictors should be removed. This also solves the issues of time (the 1950 splits) --- if there are truly intrinsic traits, then this should be timeless.

As a compromise, present the predicted probabilities as a column in Table 3.

(2) The post-1950s models contribute nothing but potential source of confusion. Omit this from main text and place in Supporting information.

(3) What can be said about species that with high introduction probability but low establishment probability?

(4) What can be said about the false negatives? if we want to follow a precautionary approach, maybe understanding these outlier profiles may be useful, too?

POINT-BY-POINT COMMENTS:

*Page 4:*

"... . However, in recent decades, the range of several mosquito vectors has expanded at an unprecedented pace. ..."

Comment : should you also mention whether this is naturally or/and in combination with human-mediated dispersal and spread?

*Page 4:*

"... These introductions have expanded the species’ ranges, sometimes into new continents and other fairway regions and enabled local disease transmission in areas considered unsuitable or free of certain diseases, a reminder that while mosquitoes can exist without transmitting pathogens, such pathogens cannot be without mosquitoes (6,7). ..."

Comment : Complex sentence. Please break this down.

*Page 4:*

"... Despite ..."

Comment : This is a very long paragraph. Break into two please.*

*Page 5:*

"... o address this question, ..."

Comment : You can break the paragraph here. Also, this new paragraph disrupts the flow of thought ---

You can perhaps put this after the objectives paragraph --- making a prediction, and this being the explanation of your predictions. OR, since it describes why specific traits were selected, maybe put this in the methods (i.e., it is a variable choice rationale).

*Page 5:*

"... In order to provide warning l ..."

Comment : You can also break the paragraph further here.

*Page 7:*

"... Table 1. ..."

Comment : I would put this as an appendix.

*Page 13:*

"... . We developed four RF models to 202 assess the invasion potential of mosquito species. The first model estimated the probability of a 203 species being introduced outside its native range, and the second the probability of the species 204 being introduced after 1950. The third and fourth models evaluated the probability of species 205 establishing themselves outside their native range (i.e., forming self-sustaining populations after 206 their introduction), considering either all records, or only those establishments occurring after 1950 207 respectively ..."

Comment : For the establishment models, did you only include species with introduction history? i.e., species with no introduction history was removed from the model?

After reading the entire paper, I do not see any value in splitting the dataset by pre-and post-1950. Remove this as this does not contribute much except for confusion.

*Page 13:*

"... Introduction and establishment dates were derived from Pabst et al. 211 (5). ..."

Comment : how many species has a history of introduction, and a history of establishment?

*Page 15:*

"... Figure 1. ..."

Comment : This is not really very relevant to the MS. So better put this in supporting information.

Alternatively, it would be better to have a diagram of your methods.

*Page 15:*

"... For each response variable, the 100 imputed datasets produced in section 2.2 were used to train 233 100 separate RF models having the same parameters, implemented in the ‘ranger’ package (49). ..."

Comment : What's missing later is how the predictions of each RF model were averaged, and variance statistics (SD) were used as a measure of uncertainty brought by the imputation process.

*Page 16:*

"... e area under the receiver operating characteristic curve (AUC; 52 ..."

Comment : How are the values of the AUC interpreted?

*Page 16:*

"... True Skill 251 Statistic (TSS; 53), ..."

Comment : How are the values of TSS interpreted?

*Page 16:*

"... Predicted probabilities were then averaged across all 100 RF model repetitions to obtain 255 stable ensemble estimates of introduction and establishment probabilities. ..."

Comment : Also mention that you also took the variance (SD) as a measure of uncertainty brought by the imputation process.

*Page 18:*

"... Overall, the establishment models slightly outperformed the introduction models, reflecting 284 greater consistency in identifying traits linked to persistence after arrival. The models correctly 285 identified 35.3 of 46 species known to be introduced beyond their native ranges and 24.1 of 29 286 established species. For the post-1950 subset, 29.8 of 41 introduced and 21.6 of 26 established 287 species were accurately predicted (Figure 2). ..."

Comment : Remove.

*Page 18:*

"... Table 2. LOOCV random forest model performance metrics (mean ± SD) based on 100 replicates predicting species 289 introduction and establishment probabilities. ..."

Comment : Place AUC at the very beginning. Also, there are so many metrics included here that were not mentioned in the methods. I suggest placing this table in the Supporting information, and only presenting a subset of the table containing the key evaluation metrics: AUC, TSS, OOB error.

I also think F1 is great to include, tho you then have to mention this in text.

*Page 19:*

"... Figure 2. ..."

Comment : I think what is most interesting here are columns B (and maybe column A, but not column C as this is already presented in the table 1).

To further complement the results text, highlight the False positives --- these are those are the potential invaders. One way to do this is giving it a red color, and the others (TP, TN, FN) are in shades of grey.

*Page 20:*

"... Results based only on ecological and life-history traits were similar in terms of 313 identified and ranking of important variables and the predicted probabilities for the introduction 314 and establishment of species ..."

Comment : Which is only the oviposition in human-made breeding sites?

*Page 20:*

"... preaders t ..."

Comment : ? you only modelled introduction and establishment.

*Page 20:*

"... were not identified by the full 316 models based on all variables (Appendix S2). ..."

Comment : Can this be a problem?

*Page 21:*

"... Figure 3. ..."

Comment : I would make 2 plots: A for both all and post 1950 introductions, the two classes separated by colors. Similarly, B for both all and post 1950 establishments, the two classes separated by colors.

This would give better contrast on how these two temporally delineated models for intro and establishemnt compared.

*Page 23:*

"... them ..."

Comment : them,

*Page 23:*

"... These include Culex vishnui, Anopheles culicifacies, Culex 345 univittatus, Aedes lineatopennis, Anopheles amictus, Culex theileri, Mansonia septempunctata, 346 Culex torrentium, Anopheles hyrcanus, Culex perexiguus, Culex poicilipes, Anopheles claviger, 347 Culex nigripalpus, Anopheles plumbeus, Culiseta inornata, Aedes geniculatus, and Anopheles 348 pseudopunctipennis. ..."

Comment : Then cite the table --- don't enumerate the scientific names here anymore.

*Page 23:*

"... These species include Anopheles rufipes, Anopheles fluviatilis, 353 Coquillettidia linealis, Coquillettidia richiardii, Anopheles sergentii, Anopheles pulcherrimus, and 354 Anopheles quadrimaculatus. ..."

Comment : Then cite the table --- don't enumerate the scientific names here anymore.

*Page 23:*

"... Eleven species with known invasion history were not captured by our introduction model 356 (predicted probabilities < 0.3). The same applies to five species in the establishment model. Most 357 of these species exhibited transient detection or failure to establish self-sustaining populations, 358 suggesting stochastic introduction events or influences from drivers beyond trait-based predictors, 359 such as chance transport or climatic anomalies. ..."

Comment : These are also informative --- what traits do they deviate in most? for a start, are they Asian species? if not, then maybe "biogeography" (as opposed to macroecology) is not a useful trait (which I think is not). I.e., is there reason to believe that only "Asian" species are more likely to be invasive? And what's with being from asia that makes them more invasive?

Or another way of thinking this is --- sure maybe historical invaders are from Asia, but with the integration of new source regions, maybe african species will be the next emerging alien species. Unfortunately, in using Asian as a variable (and was even the strongest variable), the african potential invaders won't be detected by the model.

After reading the rest of the manuscript, I realised you acknowledge this major problem. See major comments and recommendations above.

*Page 24:*

"... Table 3. ..."

Comment : "TRUE LABEL" and "PREDICTED LABEL" column doesnt make sense --- they are all nos and yes. I suggest showing predicted probabilities for for introduction and establishment for full models and traits only models.

*Page 25:*

"... 364 Occurrence of possible spreaders ..."

Comment : Can you also plot the occurrence data? becuase country boundaries are not really biogeographical.

*Page 26:*

"... his pattern 392 reflects Asia’s role as principal source region for invasive species overall (60), ..."

Comment : But is there reason to believe that they will remain a future source?

*Page 27:*

"... Europe. ..."

Comment : Paragraph break

*Page 27:*

"... integrative factor ..."

Comment : more like an integrative proxy

*Page 27:*

"... Nevertheless, our results suggest that the intrinsic traits of a species remain 418 relatively stable predictors, independent of macroecological factors (Appendix S2). ..."

Comment : They are stable, and thus they may be less biased to biogeographical source information.

*Page 28:*

"... 5,71) ..."

Comment : also 22

*Page 28:*

"... thermally extreme ..."

Comment : this can be either hot or cold.... but you only mean hot

*Page 29:*

"... n. ..."

Comment : 22

*Page 30:*

"... 24 species with no recorded 471 invasion history but trait profiles closely matching those of known invaders. ..."

Comment : What do we do with the other 7 low-establishment probability species?

*Page 31:*

"... Nonetheless, the imputation process may 490 obscure subtle ecological differences that influence invasion potential ..."

Comment : but the SD of your predictions are small. So it seems the model is not sensitive to the imputation process.

Reviewer #2: Both the research idea and the methods used to implement it are interesting. The authors rightly note that the invasion potential of mosquitoes can only be partially predicted based on intrinsic biological and ecological traits alone. However, this direction holds great potential for subsequent research and methodological refinement. It is very important that the article has practical application – it allows the findings to be used to support early warning systems, the targeted allocation of surveillance resources, and ultimately, the reduction of the risk of emerging vector-borne diseases.

Reviewer #3: I think this is a very interesting study, a large analysis with prediction of invasive potential of mosquitoes capable of transmitting human disease. Overall, I think this needs minor revision. Some areas should be clarified for what the results mean outside of the model, see comment for line 310 as example. A full list of species being assessed is needed somewhere in the paper, probably best for an appendix. I also would like the authors to explicitly list which known invasive mosquitoes didn't show as having high invasive potential from the model and describing why the model contradicts with these truly invasive mosquitoes.

**********

PLOS authors have the option to publish the peer review history of their article (what does this mean?). If published, this will include your full peer review and any attached files.

If you choose “no”, your identity will remain anonymous but your review may still be made public.

**Do you want your identity to be public for this peer review?**  For information about this choice, including consent withdrawal, please see our Privacy Policy.

Reviewer #1: No

Reviewer #2: **Yes:**  Vladimir Dubyanskiy

Reviewer #3: No

[NOTE: If reviewer comments were submitted as an attachment file, they will be attached to this email and accessible via the submission site. Please log into your account, locate the manuscript record, and check for the action link "View Attachments". If this link does not appear, there are no attachment files.]

**Figure resubmission:**

While revising your submission, we strongly recommend that you use PLOS’s NAAS tool (https://ngplosjournals.pagemajik.ai/artanalysis) to test your figure files. NAAS can convert your figure files to the TIFF file type and meet basic requirements (such as print size, resolution), or provide you with a report on issues that do not meet our requirements and that NAAS cannot fix.

After uploading your figures to PLOS’s NAAS tool - https://ngplosjournals.pagemajik.ai/artanalysis, NAAS will process the files provided and display the results in the "Uploaded Files" section of the page as the processing is complete. If the uploaded figures meet our requirements (or NAAS is able to fix the files to meet our requirements), the figure will be marked as "fixed" above. If NAAS is unable to fix the files, a red "failed" label will appear above. When NAAS has confirmed that the figure files meet our requirements, please download the file via the download option, and include these NAAS processed figure files when submitting your revised manuscript.

**Reproducibility:**

To enhance the reproducibility of your results, we recommend that authors of applicable studies deposit laboratory protocols in protocols.io, where a protocol can be assigned its own identifier (DOI) such that it can be cited independently in the future. Additionally, PLOS ONE offers an option to publish peer-reviewed clinical study protocols. Read more information on sharing protocols at https://plos.org/protocols?utm_medium=editorial-email&utm_source=authorletters&utm_campaign=protocols

# Author comment: Author response to Decision Letter 1

**DOI:** 10.1371/journal.pntd.0014538.r002

27 May 2026

Attachment Submitted filename: Response to the reviewers.pdf

# Editor report: Decision Letter 1

**DOI:** 10.1371/journal.pntd.0014538.r003

**Authors:** Paul O. Mireji, Clarence Mang’era

2 Jul 2026

Dear Mrs Pabst,

We are pleased to inform you that your manuscript 'Trait-based prediction of disease-vector mosquito invasion potential' has been provisionally accepted for publication in PLOS Neglected Tropical Diseases.

Before your manuscript can be formally accepted you will need to complete some formatting changes, which you will receive in a follow up email. A member of our team will be in touch with a set of requests.

Please note that your manuscript will not be scheduled for publication until you have made the required changes, so a swift response is appreciated.

IMPORTANT: The editorial review process is now complete. PLOS will only permit corrections to spelling, formatting or significant scientific errors from this point onwards. Requests for major changes, or any which affect the scientific understanding of your work, will cause delays to the publication date of your manuscript.

Should you, your institution's press office or the journal office choose to press release your paper, you will automatically be opted out of early publication. We ask that you notify us now if you or your institution is planning to press release the article. All press must be co-ordinated with PLOS.

Thank you again for supporting Open Access publishing; we are looking forward to publishing your work in PLOS Neglected Tropical Diseases.

Best regards,

Clarence Mang'era, PhD

Academic Editor

PLOS Neglected Tropical Diseases

Paul Mireji

Section Editor

PLOS Neglected Tropical Diseases

Shaden Kamhawi

co-Editor-in-Chief

PLOS Neglected Tropical Diseases

orcid.org/0000-0003-4304-636XX

Paul Brindley

co-Editor-in-Chief

PLOS Neglected Tropical Diseases

orcid.org/0000-0003-1765-0002

***********************************************************

The comments are substantially addressed, and the MS is greatly improved. Check the accuracy and clarity of the intended paranthesis/citation on line 67. Avoid the use of 'i.e' and 'e.g' in brackets (line 81, line 134, line 518, among others).

p.p1 {margin: 0.0px 0.0px 0.0px 0.0px; line-height: 16.0px; font: 14.0px Arial; color: #323333; -webkit-text-stroke: #323333}span.s1 {font-kerning: none

# Editor report: Acceptance letter

**DOI:** 10.1371/journal.pntd.0014538.r004

**Authors:** Paul O. Mireji, Clarence Mang’era

Dear Mrs Pabst,

We are delighted to inform you that your manuscript, "

Trait-based prediction of disease-vector mosquito invasion potential," has been formally accepted for publication in PLOS Neglected Tropical Diseases.

We have now passed your article onto the PLOS Production Department who will complete the rest of the publication process. All authors will receive a confirmation email upon publication.

The corresponding author will soon be receiving a typeset proof for review, to ensure errors have not been introduced during production. Please review the PDF proof of your manuscript carefully, as this is the last chance to correct any scientific or type-setting errors. Please note that major changes, or those which affect the scientific understanding of the work, will likely cause delays to the publication date of your manuscript. Note: Proofs for Front Matter articles (Editorial, Viewpoint, Symposium, Review, etc...) are generated on a different schedule and may not be made available as quickly.

Soon after your final files are uploaded, the early version of your manuscript will be published online unless you opted out of this process. The date of the early version will be your article's publication date. The final article will be published to the same URL, and all versions of the paper will be accessible to readers.

For Research Articles, you will receive an invoice from PLOS for your publication fee after your manuscript has reached the completed accept phase. If you receive an email requesting payment before acceptance or for any other service, this may be a phishing scheme. Learn how to identify phishing emails and protect your accounts at https://explore.plos.org/phishing.

Thank you again for supporting open-access publishing; we are looking forward to publishing your work in PLOS Neglected Tropical Diseases.

Best regards,

Shaden Kamhawi

co-Editor-in-Chief

PLOS Neglected Tropical Diseases

Paul Brindley

co-Editor-in-Chief

PLOS Neglected Tropical Diseases
